# Search where you will find most: Comparing the disciplinary coverage of 56 bibliographic databases

**DOI:** 10.1007/s11192-022-04289-7

**Published:** 2022-05-06

**Authors:** Michael Gusenbauer

**Affiliations:** University of Innsbruck, Innsbruck, Austria

**Keywords:** Subject coverage, Comparison, Bibliographic database, Search system, Basket of keywords, Query hit counts

## Abstract

This paper introduces a novel scientometrics method and applies it to estimate the subject coverages of many of the popular English-focused bibliographic databases in academia. The method uses query results as a common denominator to compare a wide variety of search engines, repositories, digital libraries, and other bibliographic databases. The method extends existing sampling-based approaches that analyze smaller sets of database coverages. The findings show the relative and absolute subject coverages of 56 databases—information that has often not been available before. Knowing the databases’ *absolute* subject coverage allows the selection of the most comprehensive databases for searches requiring high recall/sensitivity, particularly relevant in lookup or exploratory searches. Knowing the databases’ *relative* subject coverage allows the selection of specialized databases for searches requiring high precision/specificity, particularly relevant in systematic searches. The findings illustrate not only differences in the disciplinary coverage of Google Scholar, Scopus, or Web of Science, but also of less frequently analyzed databases. For example, researchers might be surprised how Meta (discontinued), Embase, or Europe PMC are found to cover more records than PubMed in Medicine and other health subjects. These findings should encourage researchers to re-evaluate their go-to databases, also against newly introduced options. Searching with more comprehensive databases can improve finding, particularly when selecting the most fitting databases needs particular thought, such as in systematic reviews and meta-analyses. This comparison can also help librarians and other information experts re-evaluate expensive database procurement strategies. Researchers without institutional access learn which open databases are likely most comprehensive in their disciplines.

## Introduction

Researchers rely on searches to be comprehensive to identify the most scholarly records relevant to their work. More than ever, researchers need to know how and where to search, predominantly because of four trends: First, exponential increases in the output of scholarly records require scholars to think where they can best access these records (Gusenbauer, 2021). Second, an increasingly diverse search system landscape makes it increasingly difficult to select the best databases and systems with which scholarly records are accessed. Most scholars still rely on Google Scholar for many of their search needs (Gusenbauer, 2021; Nicholas et al., 2017). Third, conduct guidance for specific scientific methods—particularly in the field of evidence synthesis—requires increasingly higher levels of rigor in identifying scholarly records (e.g., Higgins et al., 2020; Kugley et al., 2016). Fourth, institutional resources available to researchers only show a partial picture of the entire range of bibliographic databases available—often the most suitable databases remain hidden behind paywalls without the knowledge of researchers. Because of these trends, researchers need guidance on which bibliographic databases are best in their discipline.

In general, two criteria determine the optimal search system for a given purpose: the search functionality offered and the coverage provided. *Search functionality* describes a searcher’s options when accessing the records on a database, such as query building, filtering, citation searching, or a controlled vocabulary. In a review of search functionalities, we have demonstrated the variance of systems’ search functionalities (Gusenbauer & Haddaway, 2020) and their importance for particular search types (Gusenbauer & Haddaway, 2021). *Coverage*, is the second important criterion and describes the number of (potentially) relevant records a system provides. There are different types of coverage: subject coverage, retrospective coverage, geographical coverage, language coverage, journal coverage, etc. For researchers knowing the subject coverage of a scholarly database is likely to be key in determining its suitability for subsequent search steps. Knowledge of subject coverage informs a researcher of the number of records on a specific subject or discipline a database hosts. A health scientist, for example, will be interested in systems with high coverage in medicine, dentistry, or nursing and low coverage of irrelevant subjects. The higher the coverage, the more relevant articles can be identified. At the moment, researchers have various ways of learning about the subject coverage of systems:

First, the database owners use textual descriptions to delimit subject coverage (see Tables 1, 2, 3, 4). These are, in most cases, severely outdated and only provide information at the coarsest level of granularity. For example, the database of EconLit is described by EBSCOhost (2021c) as “the American Economic Association's electronic database, is the world's foremost source of references to economic literature. The database contains more than 1.1 million records from 1886 to present. EconLit covers virtually every area related to economics.” At the time of writing, EconLit covers more than 1.8 million articles and covers topics related to Business and Social Sciences in addition to Economics. Outdated and inaccurate descriptions are not the exception, but the rule, and can be found particularly across aggregator providers (EBSCOhost, Ovid, ProQuest, Web of Science). Most databases and search systems only provide rudimentary information on subject coverage, and some do not provide any (e.g., Google Scholar or scite).

Second, database providers give some lists of journal coverage, so authors can verify if and to what extent particular journals are covered by a database, albeit that information is not always current. While the information is precise at the journal level, it is also inconvenient as these lists are not always available. Furthermore, researchers might be interested in the coverage of an entire discipline rather than in single journals. Researchers do not get a clear picture of how well one database compares with others.

Third, because of the great relevance of coverage as a decisive factor for optimal database choice, a considerable number of scientometrics studies analyze the coverage of databases. Some analyze single databases (Hug & Braendle, 2017), some compare pairs (Chadegani et al., 2013; Mongeon & Paul-Hus, 2016; Moya-Anegón et al., 2007), triples (García-Pérez, 2010; Harzing & Alakangas, 2016; Martín-Martín et al., 2018a; Singh et al., 2021), or up to five or six databases (Harzing, 2019; Martín-Martín et al., 2021; Visser et al., 2021). Next to the number of databases that are compared, these studies differ in which data they analyze. Some analyze databases via journals (Harzing, 2019; Mongeon & Paul-Hus, 2016; Moya-Anegón et al., 2007; Singh et al., 2021) or individual records (Vera-Baceta et al., 2019) and their citations (Martín-Martín et al., 2018a). The goal is to assess language coverage (Vera-Baceta et al., 2019), geographic coverage (Singh et al., 2021), record type coverage, subject coverage, (Martín-Martín et al., 2018a; Meho & Yang, 2007), journal coverage, citation coverage (García-Pérez, 2010) or a mix of those (Harzing, 2019; Harzing & Alakangas, 2016; Moya-Anegón et al., 2007; Singh et al., 2021; Visser et al., 2021).

One recent study was particularly influential in assessing the overlap of subject categories between the databases of Google Scholar, Web of Science Core Collection (WOS CC), and Scopus (Martín-Martín et al., 2018b). Its results show differences in the degree to which databases cover records from specific subjects. The analysis was novel as the dataset of citations was particularly comprehensive and the data were presented at multiple levels of granularity of the underlying Google Scholar subject classification. The authors followed that paper with a similar method expanding the databases analyzed to include Microsoft Academic, Dimensions, and OpenCitations’ COCI (Martín-Martín et al., 2021). Both studies are informative on the overlap of databases regarding a specific sample of records, in this case, highly-cited documents.

Nevertheless, when researchers want to know the subject coverage of an *entire* database, sampling-based methodologies have some limitations. A sample, even when it covers as many as 2.4 million (Martín-Martín et al., 2018a) or 3.1 million citations of highly-cited documents (Martín-Martín et al., 2021), is likely to inaccurately assess the subject coverage of databases whose total coverage are more than 254 million (Microsoft Academic) or more than 389 million records (Google Scholar) (Gusenbauer, 2019). Deriving an even more comprehensive sample is difficult, as database providers are highly protective of their bibliographic data. Extracting data from databases, most of which restrict downloads by various measures makes scientometric analyses strenuous and the burden increases in parallel with the number of databases investigated. Accordingly, current estimations of databases’ subject coverage are limited to specific document types and are limited in the number of databases they analyze.

This study takes a new methodological route of analyzing, estimating, and comparing subject coverage across databases—an approach necessary to allow the assessment of overall subject coverage for a large number of databases. It applies the method of query hit counts (QHC) used in scientometric analyses (e.g., Da Teixeira Silva et al., 2020; Gusenbauer, 2019; Kousha & Thelwall, 2020; Lazarus et al., 2020; Orduña-Malea et al., 2015) to determine subject coverage. The QHC method is particularly beneficial as it allows access to the entire database without requiring the download of individual records. Furthermore, the QHC method can assess many systems, with high reproducibility, at relatively low marginal cost, and with a relatively high level of precision (see validation of results). The method’s compatibility allows the relatively straightforward addition of new systems to the analysis and for subject coverage information to be rapidly updated. As the methodological considerations are novel, this study describes them in detail; first addressing the methodological consideration in general, and second the detailed analytical steps taken.

This study presents detailed results on the absolute and the relative coverage of 56 academic databases and discusses the precision of estimates and how the results can be applied in research practice. It also discusses these results in light of the limitations inherent to the new method. An overview of the central concepts used throughout this study can be found in the Appendix.

## Methodological considerations

The foremost goal of this study is to estimate the number of records available on a database relating to a specific subject. Knowing the absolute number of records on a subject available on a database can be used to calculate its relative share of all records. Both the absolute and the relative number of subject-attributable records on a database are key criteria for optimal database selection.

This study applies a new way of assessing subject coverage. The procedure can be seen as a new method to be added to existing sampling-based methods. Unlike existing methods, I propose and rely on carefully selected keywords to estimate the subject coverage of databases. Below I list the fundamental assumptions underlying this new method:

- Keyword-based queries are universally available across scholarly databases and are by far the most common way of accessing scholarly records. Only a few bibliographic databases provide no query-based search options.
- Information based on query hit counts—the number of records identified with a specific keyword-based query—is generally available and mostly accurate.
- Searching with a specific keyword will retrieve a set of records that contain the keyword in a specified body of text. This makes it possible to determine coverage, even without downloading the individual records. Knowing the number of hits makes it possible to assess the contents of a database.
- Keyword-based queries are an effective way of accessing *all* records available on a database (method see e.g., Gusenbauer, 2019). Extracting samples of records is not necessary.
- Each record stored in a scholarly database is attributable to one or multiple subject categories from a general science classification system (e.g., the All Science Journal Classification).
- Keywords are more or less subject-specific, that is, keywords are more or less attributable to a specific subject category. Some keywords are more clearly attributable to a single subject category than others. The degree of attributability is a keyword’s degree of specificity: its keyword precision.
- If a database has high coverage of records identifiable with specific keywords, then the subjects associated with these keywords will also be highly prevalent on that database. If a database search does not retrieve records for certain subject-specific keywords, it is unlikely that it will contain records from that subject.
- The more subject-specific keywords estimate the subject coverage of a database, the more reliable the resulting estimate. Marginal rates of additional precision decrease with the number of keywords.
- The capacity of a keyword to identify records from a specific subject, that is, its precision and recall, is the same across databases, if the underlying databases and search functionalities are the same. Under these conditions, a keyword will *accurately* estimate subject coverage across databases and systems. If differences in either one or both the database or the search functionalities occur, the keyword will estimate subject coverage less accurately.
- To derive a sufficiently accurate estimate of subject coverage across databases, the keyword-based method requires a universal approach that holds as many aspects of search functionalities and database composition constant.
- Primarily, two kinds of biases must be accounted for, mostly by selecting the types of search systems to which the method is applied. First, biases are introduced by differences in search functionality, mostly through inaccurate query interpretation and the unavailability of comparable field codes. Second, biases in the method are introduced by differences in the nature of the records available at the estimated databases, most importantly in terms of language and record type.

Having outlined the fundamental assumptions of the method, the following explains the detailed consideration of the keyword-based logic, query hit counts, the classification system, accuracy assessment, database, and keyword selection.

### Basket of keywords (BOK)

The logic the keyword-based method employs can be described as a basket of keywords (BOK). The term BOK is inspired by economics, where a basket of goods is used to determine the consumer price index, a vehicle to determine inflation (Bryan & Cecchetti, 1993; OECD, 2021). The consumer price index is estimated by selecting different goods that are more or less regularly purchased by consumers. The variation in prices of those goods determines the overall inflation. Similarly, the BOK approach uses selected keywords and analyzes various databases using the same method. As inflation can be compared across countries and time, the BOK approach allows comparisons across databases at various points in time.

Just as the basket of goods only uses selected goods, the basket of keywords also relies on a representative selection of keywords: In this study, 14 keywords for each of the subject categories. As the 14 keywords are a selection of the most distinctive terms used in the subject category, the estimate based on these keywords is valid to determine overall subject coverage. Just as the inflation score assumes that overall prices increase when the prices of *bread* and *butter* increase, the BOK logic assumes high subject coverage in Physics and Astronomy if the database has many hits for the keywords *boson* or *quark*. The likelihood of an accurate coverage estimate increases with the number of subject-specific keywords that estimate a similarly high coverage.

### Query hit counts (QHC)

The BOK approach does not use the crude query hit count (QHC) data to estimate overall coverage but relies on the keywords’ representativeness to access all records from a specific subject; in other words, its recall. For example, a title search with the term *boson* identifies 0.22% of all records from Physics and Astronomy at Scopus, the database chosen to be the representative database. Each QHC of a keyword will provide an estimate of the subject coverage of a system. For example, a title search for *boson* identifies 12,329 hits at WOS CC (indexes see Table 2), estimating the size of the total coverage of Physics and Astronomy at 5.5 million records. This is one of many estimates used as input to determine the overall subject coverage of databases. Having addressed the problem of a record having multiple subjects, the exact method will be explained in more detail in the analytic steps section.

While this study is the first to compare a large set of bibliographic databases with the BOK method, the underlying QHC method is not new. It has been applied to estimate or determine, for example, the sizes of bibliographic databases (Gusenbauer, 2019), determine the coverage of Covid-19 literature (Da Teixeira Silva et al., 2020; Kousha & Thelwall, 2020; Lazarus et al., 2020) and to estimate institutions’ contributions to UN sustainable development goals (Jayabalasingham et al., 2019). Because of the great accessibility of data (compared to the other sampling methods), the BOK approach allows the comparison of a great number of databases.

### Selection of reference database and its subject classification: Why ASJC by Scopus?

In determining the subject coverage of each database, one of the biggest decisions was the choice of subject classification system. There are numerous systems classifying scientific records into the known science disciplines. They differ in depth (number of hierarchies), the levels of granularity (number of categories at a level of hierarchy), level of multi-attribution (the number of subjects attributed to a single record), and scope (all science vs. subfields of science).

The two main classification systems used in research and often compared are the WOS classification and Scopus All Science Journal Classification (ASJC) (Franceschini et al., 2016; Wang & Waltman, 2016). Those systems are the most prominent because the databases to which they are mainly applied are central in research discovery, and accordingly, researchers rely on the subject filters based on those classifications. Next to WOS and ASJC, other prominent classification systems include OECD’s Field of Science and Technology (FOS) (OECD, 2007), or the Dewey system that was continuously replaced by the Library of Congress classification used in libraries (Shorten et al., 2005). These classification systems mostly rely on manual curation mechanisms at the journal level to classify records (Waltman & van Eck, 2012). With new computational methods, new classification approaches became available. Microsoft Academic Graph for example classifies records directly with its Fields of Study system that includes 713,888 different machine-generated topics at different levels (Shen et al., 2018). This classification is also adopted by Dimensions, but the accuracy and completeness of the approach have been questioned (Bornmann, 2018; Herzog & Lunn, 2018; Orduña-Malea & Delgado-López-Cózar, 2018; Visser et al., 2021).

Classification systems are not perfect. While WOS classification and ASJC have high standards in data curation and classification, they are also criticized for misclassifying records (Shu et al., 2019), the journal-level classification, and a lack of transparency (Wang & Waltman, 2016). One of the limitations of ASJC is that it assigns categories to records quite liberally, and consequently, many records are assigned to multiple disciplines.

There is no overarching standard in classification systems. Various systems are used and there is little compatibility between them that would permit straightforward comparison. Accordingly, it was important that this study adopted a single classification system used throughout to guarantee comparability across databases. The decision of which system that should be was based on both the classification system and the system and database through which it was *analyzable*. Accordingly, in choosing the best system, four main aspects had to be accounted for: First, the classification system should be rolled out over all records in a large-scale, all-science database, and the classification information should be retrievable from its search interface. Second, the classification system should possess an intermediate level of granularity; meaning it should be sufficiently coarse to limit the number of categories serving this analysis and fine enough to be useful as a decision tool for researchers. Third, the search system that employs the classification had to be searchable through some widely used field codes such as title, abstract, and an ‘all fields’ field code. That degree of searchability permits comparability between data in the consecutive analysis. Fourth, the search system should have some history that would indicate its likely continuity (notable exceptions are the discontinued Microsoft Academic and Meta).

The above criteria determined that the only two candidates were WOS classification and ASJC. After careful consideration and testing of both systems, ASJC was chosen over WOS classification, not because it is the best classification system *overall* (see e.g., Wang & Waltman, 2016), but because it is the best classification *for the purposes of this study.* The main reason was the level of classification granularity available at the search interface. In 2021 Scopus provided ASJC information at the 26 (+ 1 multidisciplinary) category level (see Table 1 and Elsevier 2022b), compared to the 254-category level at the WOS interface (for detailed information see Clarivate Analytics, 2022b). At its most granular level, the WOS classification system updates frequently adding new categories: last from 252 to 254 categories months before data collection in 2021. These regular updates are advantageous as it keeps the categories relevant, reflecting changes in research practice. Yet, to ensure comparability of the collected data over time, a more consistent classification system/level is preferable. Thus, for the consecutive analysis, the 26-category ASJC system was considered a better choice, mainly in terms of its intermediate granularity limiting the number of keywords needed to determine subject coverage and because of its superior consistency—the 26-category system was already mentioned by Flanagan (2014), after it updated from a 25-category system.

Scopus’ ASJC classification has many merits, particularly compared to its peer and rival the Web of Science classification (for a comparison, see Wang & Waltman, 2016). While Scopus, has slightly better coverage than WOS CC (for the versions available to me—see Table 2), this was only marginally relevant for the selection of Scopus/ASJC (Gusenbauer, 2019; Mongeon & Paul-Hus, 2016). A more important argument for Scopus as the reference system of choice is that it does not differentiate in institutional coverage packages, while WOS offers tailored database packages, which complicates comparison. This lack of comparability of WOS Core Collection (WOS CC) is a problem neglected by too many scholars, even those involved in scientometrics research. Too many researchers treat WOS as a homogeneous database yet forget that even its most popular product, the Core Collection, differs substantially in coverage across institutions. For example, in 2021 the University of Innsbruck’s WOS CC access covered 79 million records, the Technical University of Munich’s only a little below 71 million. Looking more closely, while the University of Innsbruck includes the Science Citation Index Expanded from 1900 to the present, the Technical University of Munich only covers it from 1945. Most other indices differ across institutions that subscribe to WOS. This is a major comparability and replicability issue that is regularly overlooked. It particularly harms the relevance of the many studies comparing coverage of WOS and its premier product, the Core Collection.

**Table 1:** The 26 subjects according to the ASJC (+ the excluded multidisciplinary subject)

Subject areas (4)	Subject area classifications (or 'Subjects') (26)	Abbreviation
Physical sciences	Chemical Engineering	CENG
	Chemistry	CHEM
	Computer Science	COMP
	Earth and Planetary Sciences	EART
	Energy	ENER
	Engineering	ENGI
	Environmental Science	ENVI
	Materials Science	MATE
	Mathematics	MATH
	Physics and Astronomy	PHYS
Life sciences	Agricultural and Biological Sciences	AGRI
	Biochemistry, Genetics and Molecular Biology	BIOC
	Immunology and Microbiology	IMMU
	Neuroscience	NEUR
	Pharmacology, Toxicology and Pharmaceutics	PHAR
Social Sciences and Humanities	Arts and Humanities	ARTS
	Business, Management and Accounting	BUSI
	Decision Sciences	DECI
	Economics, Econometrics and Finance	ECON
	Psychology	PSYC
	Social Sciences	SOCI
Health sciences	Dentistry	DENT
	Health Professions	HEAL
	Medicine	MEDI
	Nursing	NURS
	Veterinary	VETE
Multidisciplinary	Multidisciplinary—not included	–

### Selection of control dataset and its classification system: WOS classification

Another reason for using the less granular ASJC classification over WOS classification as the reference system was the option to *compare* the 26 ASJC categories with the 254 WOS categories, but not the other way around. Matching both classification systems makes it possible to estimate the subject coverage of WOS in terms of ASJC classification.

While both systems have three different levels of granularity, only one level of granularity is available in the search interface of the systems—the other levels are not available for assessing subject coverage and are thus irrelevant here. Therefore, ASJC was chosen as the *reference* dataset, and WOS classification was used to create a *control* dataset to verify the accuracy of the estimation of WOS subject coverage via the ASJC systems. The steps taken to determine estimation accuracy via comparing WOS and ASJC are described in the chapter ‘validation of estimates’.

### Selection of databases for estimation of subject coverage

This study focused on determining subject coverage of the largest number of databases deemed popular or important in research (see Table 2). The *type* of search system—the system that makes records on a database accessible—was not relevant when selecting the databases to investigate. It did not matter whether a search system was a search engine, a repository of some kind, a bibliographic database, a digital library, a journal platform, or an aggregator. However, the type of records a database covered and some functionality criteria were relevant. Hence, only databases that were active and accessible, had a minimum of 1 million records, (likely^1^) a majority of English records, and that were reasonably popular among researchers were selected. The goal was to include all large multidisciplinary databases (i.e., Google Scholar, Microsoft Academic, Scopus, WOS CC, Mendeley, Crossref, ScienceDirect, JSTOR), particularly the newer ones (i.e., Lens, Dimensions, scite, Meta, Semantic Scholar), databases focused on open access records (i.e., Core, BASE, arXiv, DOAJ, Paperity, OpenAIRE), A/B-rated journal platforms ranked in the WASS-SENSE ranking 2020 (e.g. SpringerLink, Wiley, SAGE), databases with high disciplinary relevance (e.g., ACM Digital Library, Medline, Europe PMC, Embase, APA PsycInfo) and all applicable databases of the previous Research Synthesis Methods paper examining search functionalities of popular systems (Gusenbauer & Haddaway, 2020).

Another selection criterion was the disciplinary focus of a database. The BOK method’s reliability would decrease if a database mainly covered only a small fraction of one of the 26 ASJC subjects. For example, if a system focuses on theater studies alone—a sub-discipline of Arts and Humanities—the keywords would not be likely to reflect its coverage reliably. To explore the issue of narrow subject focus of the underlying data, this study included two popular databases from education research (ERIC) and sports health (SPORTDiscus).

Medline, APA PsycInfo, and ERIC were included multiple times via different database providers, to validate the BOK method and to see whether database coverage differed. The reason for including a large number of popular databases was to help readers decide which databases cover the most records in their discipline and demonstrate the merits of the BOK method. Data were collected between June and August 2021 via tens of thousands of manual queries (even low marginal cost accumulates to considerable work…).

The following Table 2 lists a total of 56 databases included (not included databases are listed here^2^). The table illustrates database type, absolute database size (ADS), retrospective coverage, record types, English coverage, and openness of the databases. It also shows the subject descriptions provided by the database providers.

**Table 2:**
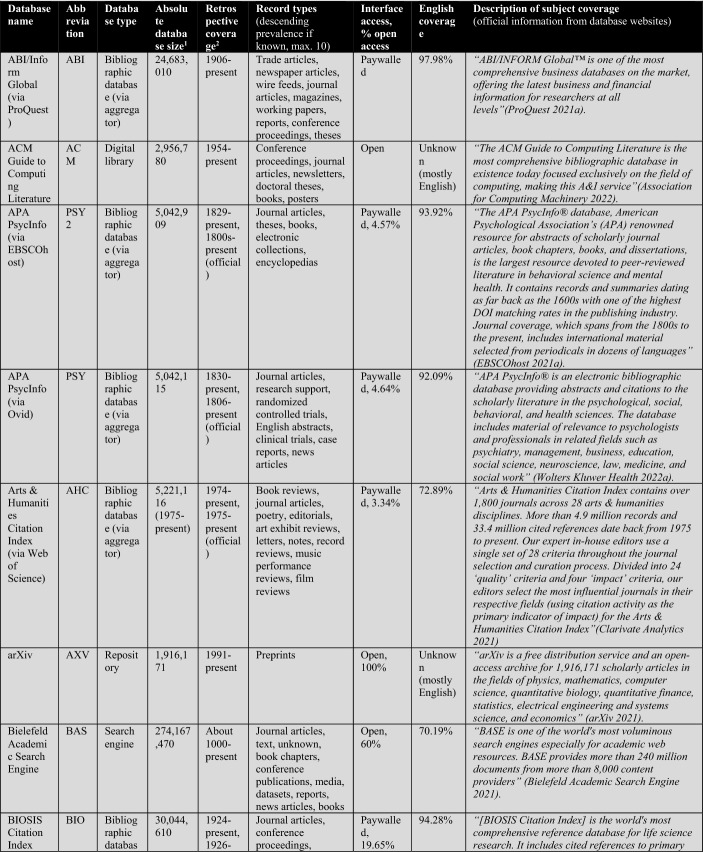

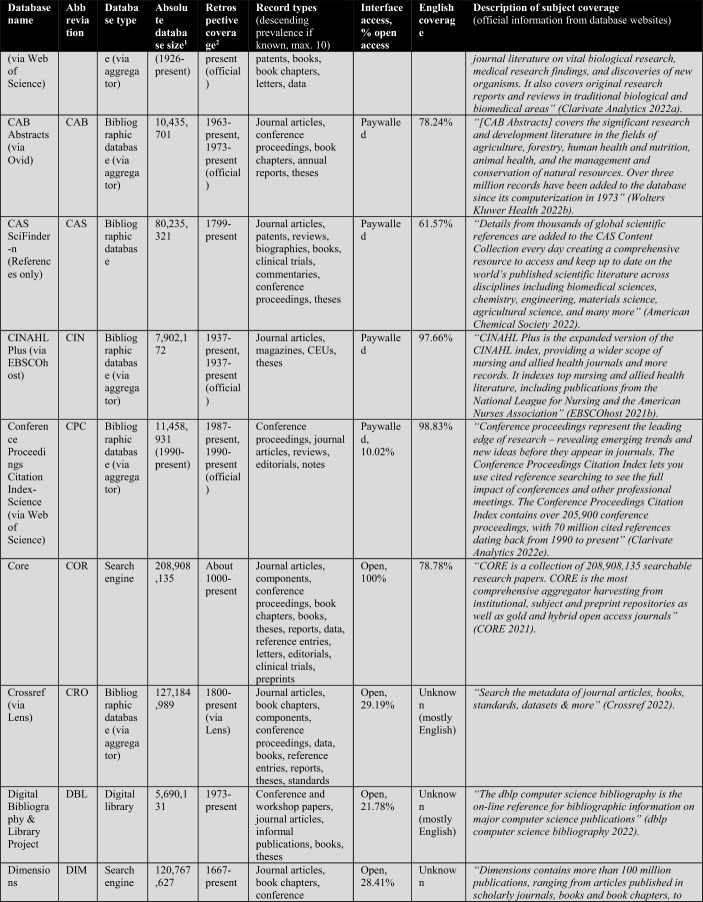

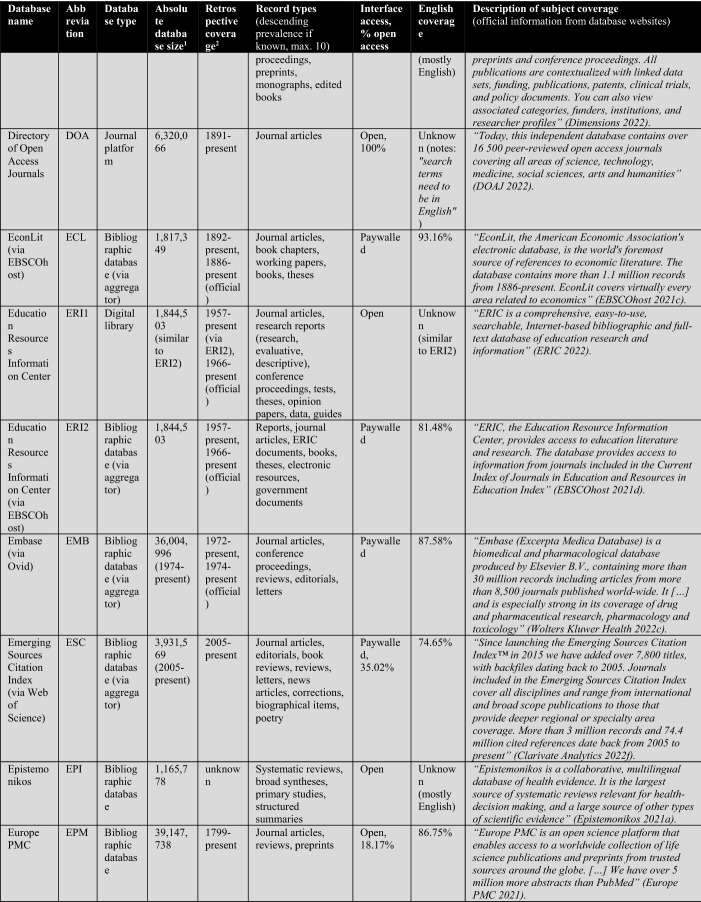

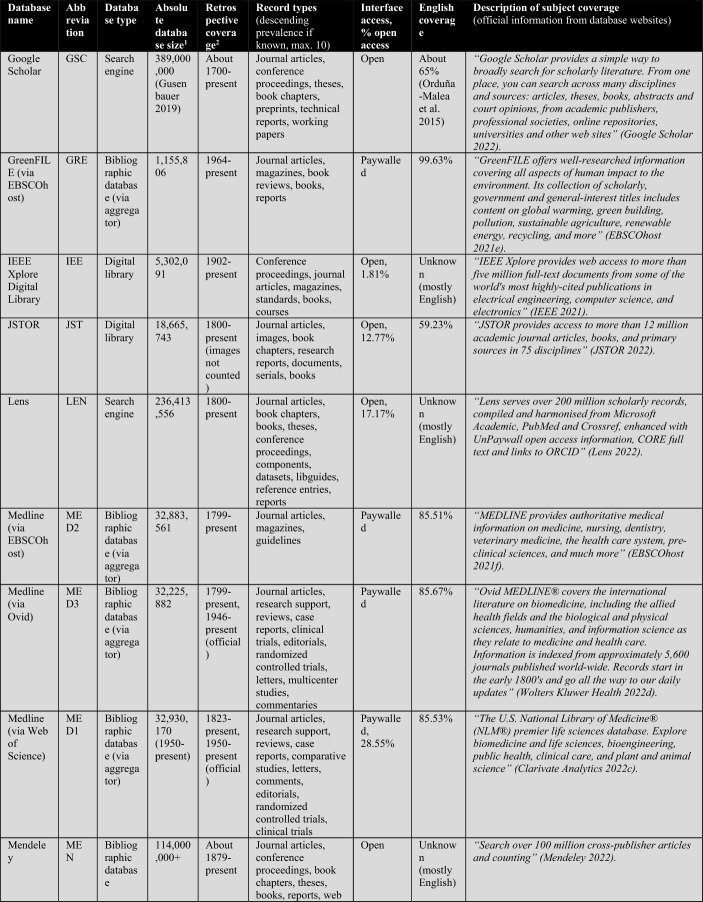

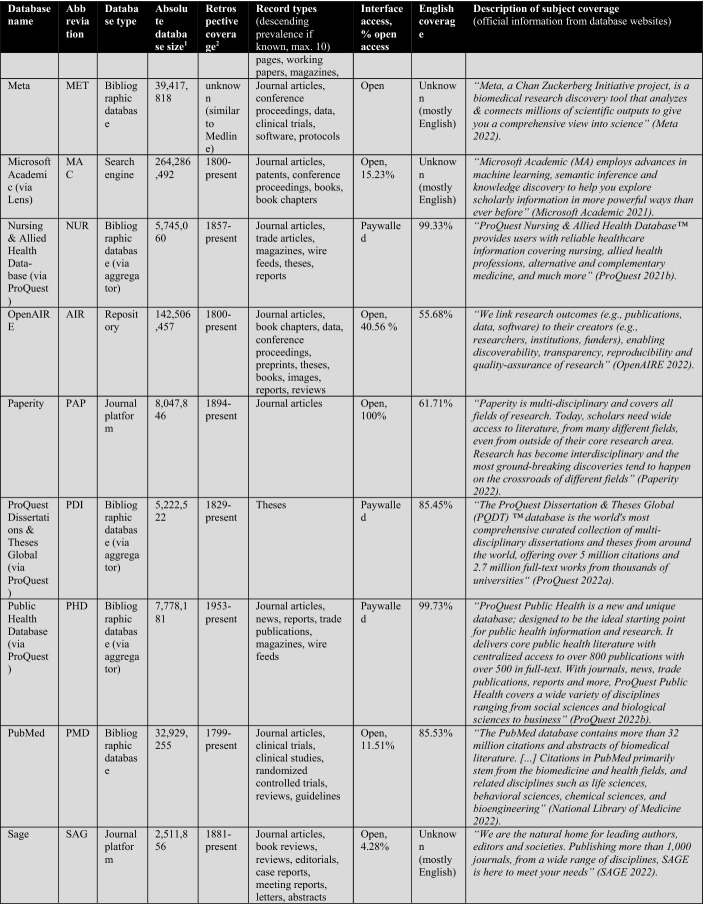

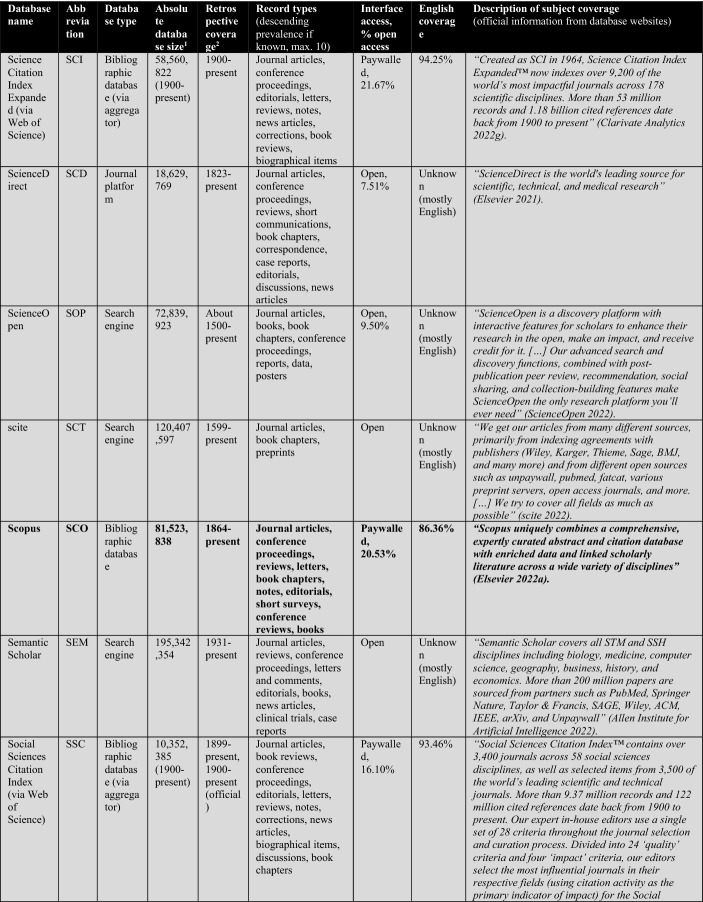

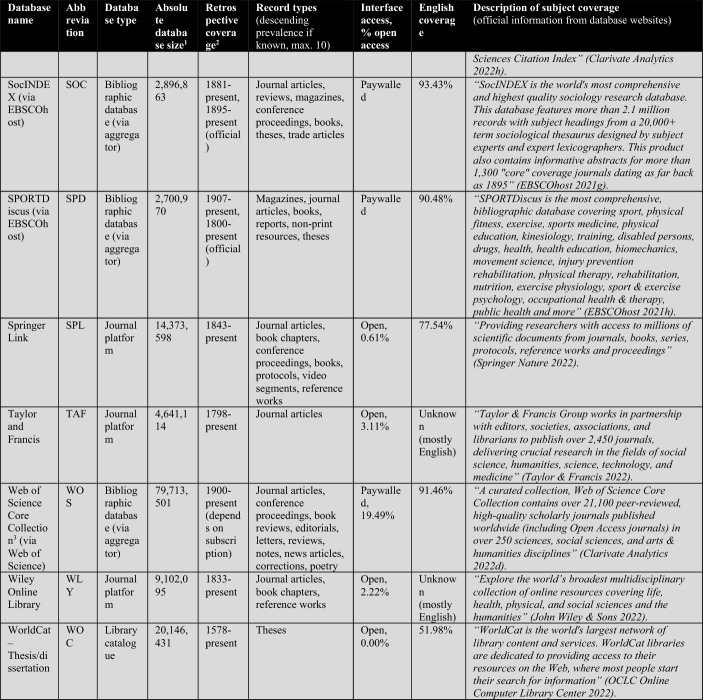
Databases analyzed for subject coverage

Abbreviations of the 56 databases:

**Table:** 

ABI = ABI/Inform Global (via ProQuest) ACM = ACM Guide to Computing Literature AHC = Arts & Humanities Citation Index (via Web of Science) AIR = OpenAIRE AXV = arXiv BAS = Bielefeld Academic Search Engine BIO = BIOSIS Citation Index (via Web of Science) CAB = CAB Abstracts (via Ovid) CAS = CAS SciFinder-n CIN = CINAHL Plus (via EBSCOhost) COR = Core CPC = Conference Proceedings Citation Index- Science (via Web of Science) CRO = Crossref DBL = Digital Bibliography & Library Project DIM = Dimensions DOA = Directory of Open Access Journals ECL = EconLit (via EBSCOhost) EMB = Embase (via Ovid)	EPI = Epistemonikos EPM = Europe PMC ERC1 = ERIC ERC2 = ERIC (via EBSCOhost) ESC = Emerging Sources Citation Index (via Web of Science) GRE = GreenFILE (via EBSCOhost) GSC = Google Scholar IEE = IEEE Xplore Digital Library JST = JSTOR LEN = Lens MAC = Microsoft Academic MED1 = Medline (via Web of Science) MED2 = Medline (via EBSCOhost) MED3 = Medline (via Ovid) MEN = mendely MET = Meta NUR = Nursing & Allied Health Database (via ProQuest) PAP = paperity PDI = ProQuest Dissertations & Theses Global	PHD = Public Health Database (via ProQuest) PMD = PubMed PSY1 = APA PsycInfo (via Ovid) PSY2 = APA PsycInfo (via EBSCOhost) SAG = Sage SCD = ScienceDirect SCI = Science Citation Index Expanded (via Web of Science) SCO = Scopus SCT = scite SEM = Semantic Scholar SOC = SocINDEX (via EBSCOhost) SOP = ScienceOpen SPD = SPORTDiscus (via EBSCOhost) SPL = SpringerLink SSC = Social Sciences Citation Index (via Web of Science) TAF = Taylor and Francis WCC = Web of Science Core Collection WLY = Wiley Online Library WOC = WorldCat-Thesis/dissertation

^a^Absolute database sizes, English and open access coverage were determined via QHCs, meta data and official information.

^b^Official coverage information, or starting year was defined as the year a database first covers at least 100 records and the year is followed by another one with also at least 100 records. Spurious coverage of single records prior to that year was not counted. Notice records from periods earlier than 1800 often have a high share of records with faulty publication year meta-data. If there was a discrepancy between official retrospective coverage reporting and actually available records, I stated both data.

^c^*INCLUDED INDEXES OF WOS CC:* Science Citation Index Expanded (SCI-EXPANDED)–1900-present *[full coverage];* Social Sciences Citation Index (SSCI)–1956-present *[max coverage would be from 1900; Δ about 700,000 records];* Arts & Humanities Citation Index (A&HCI)–1975-present *[full coverage];* Emerging Sources Citation Index (ESCI)–2005-present *[full coverage];* Conference Proceedings Citation Index-Science (CPCI-S)–1998-present *[max coverage would be from 1990; Δ about 1,500,000 records];* Conference Proceedings Citation Index-Social Science & Humanities (CPCI-SSH)–1998-present *[max coverage would be from 1990; Δ about 200,000 records]*

*NOT AVAILABLE INDEXES OF WOS CC:* Book Citation Index (BKCI-S/SSH)–2005-present *[Δ about 1,500,000 records];* Current Chemical Reactions (CCR-Expanded)–1985-present *[Δ about 300,000 records];* Index Chemicus (IC)–1993-present *[Δ about 500,000 records]*

### Selection of the keywords in the basket

To determine the basket of keywords used to estimate subject coverage, I first tried subject description terms, such as *Social Sciences* or *Theoretical Computer Science*. However, I found that the terms had to be as simple as possible to maximize comparability across systems that all varied in search functionalities. Accordingly, I chose unigrams, not bigrams. This chosen method meant I could reduce the likelihood of misinterpretation by single search systems. Even though bigrams are more individual and would permit higher levels of precision, unigrams were chosen due to their higher compatibility.

#### Query subject counts (QSCs) to determine keyword precision

With a preliminary list of keywords, I tested the effectiveness of keyword queries retrieving accurate estimates of subject coverage. It became clear that the precision of keywords was the most critical determinant. The precision of keywords was determined via the query *subject* counts (QSC), which capture the subject-precision of a keyword at the reference system Scopus. Based on the ASJC classification system available at Scopus, each keyword query retrieves a set of 26 QSCs, denoting the number of records identified for each subject category. The precision of a keyword is determined by dividing the QSC of a subject by the sum of QSCs for that subject. Each keyword is more or less representative of a subject, being reflected by the precision value of the keyword for the specific subject.

Ideally, the subject-precision of a keyword would be most constant over search systems and databases—i.e., a keyword should always be similarly representative of a subject. However, keywords always run the risk of being ambiguous and associated with different subject attributes at different databases. An extreme case would be, for example, “jaguar” being an animal or a vehicle manufacturer. The keyword would deliver different estimates depending on whether the database is focused on biology or manufacturing. Accordingly, I took care to select the least ambiguous keywords, that is to say, the keywords with the greatest level of precision in a single subject category. An example is “evolution”, which is relatively unsuitable as it is not only used in biology but also refers to progress in general. While precision can be as low as 0, the highest precision was identified in title searches for “branes”, a keyword used almost exclusively (in 90.3% of records) in Physics and Astronomy. The keyword almost unequivocally refers to objects associated with String Theory, meaning almost no other subject uses the term. Accordingly, the likelihood of accurately estimating the Physics and Astronomy coverage of a system with this keyword is high. While QSCs were captured only at the reference database (Scopus), the number of hits a keyword retrieved (QHCs) were captured at all other databases.

#### A systematic approach for selecting keywords

For each of the 26 subject categories from the ASJC classification, 14 keywords were identified to be used as input to estimate the subject coverage of databases. To maximize the precision of the selected keywords, I needed a systematic approach to compare promising subject-specific keywords and select the most suitable ones. Accordingly, I downloaded 6000 publication records uniquely attributed to each of the 26 subjects from Scopus (a total of 156,000 records). For each subject, I selected the 2000 most cited records for the three time periods of 1991–2000, 2001–2010, and 2011–2020. I downloaded only records attributed solely to the subject in question and that had no overlaps with other subjects (no multi-attribution). As tests showed that title-only searches yielded the most precise estimates, keyword selection was also based on the title field. I calculated term frequencies (tf) and inverse document frequency (idf) for the 156,000 record titles to assess the prevalence and uniqueness of the keywords. The keywords chosen were those least shared *across* subjects, but which were highly prevalent *within* a subject. The focus was to identify the most unique terms to determine subject coverage of databases. The more unique a term is (i.e., it is a technical term only used in a specific subject context), the better it is for estimating. Accordingly, I ranked the list of keywords with the following formula: tf × idf^5 that favored uniqueness over prevalence. Keywords were only selected when they were unigrams, no numbers, no terms with three characters or fewer, and no special characters. For a list of the first 60 to 120 keywords, I retrieved QSC data for each of the keywords from Scopus.

To determine the BOK most suitable for the consecutive analysis of databases, I needed to determine their precision and recall of keywords used to determine subject coverage. Precision is important, as precise keywords associate records to subjects most unambiguously. Recall is important, as keywords used too infrequently might lead to biased estimates. Pre-tests showed that precision, rather than recall, determined the accuracy of the overall subject estimation and I thus gave more weight to precision in selecting the 14 keywords for each subject. Based on the data, I ranked the keywords with the following formula: recall × precision^5. As a result, I collected 14 keywords for each of the 26 subjects resulting in a list of 364 keywords (see “Appendix”).

## Analytic steps

The analysis section describes the steps necessary to calculate a plausible and precise estimate of subject coverage from single QHCs. A robust method was arrived at with the help of some iterations and a control database to assess changes in the accuracy of estimates. The method is suitable for determining precise estimates of subject coverage at 56 bibliographic databases. In general, the goal was to create an accurate, yet robust model, that is, one that was the least database-fitted. The reasons for the methodological choices are described in detail in this section.

### Step 1: determination of recall and precision values based on Scopus and ASJC

To derive the first estimates of subject coverage, I needed to determine each keyword’s capacity to estimate subjects. This assessment was based on the Scopus database and its ASJC classification system, where each keyword query retrieves a set of records attributable to several subjects—the query subject counts (QSCs). I collected information on the recall and precision of each keyword. Recall was determined by the share of records attributed to a subject from all records in the Scopus database on the subject. The recall measure reveals that, for example, of all the records from Physics and Astronomy, 0.38% have “neutrino” in their title. Precision was determined by the proportion of records attributable to a specific subject from the sum of all subject attributions for this keyword, or the QSC for a specific subject divided by the QSC for all subjects. The precision measure reveals that, for example, of all the records with “neutrino” in the title, 81.4% are attributable to Physics and Astronomy. If a keyword identified more records from a specific subject, its recall was higher. If it identified more records from one subject, and fewer from other subjects, its precision was higher. As a result, I had 28,392 recall and precision values derived from 364 keywords, for each of the 26 subjects, for each of the three field codes considered: ‘title’, ‘abstract’ and ‘all fields’. These values were all based on Scopus’s ASJC classification of about 82 million scholarly records in July 2021.

The fact that the Scopus dataset was based on records that are attributed to one or multiple subjects (multi-attribution) needed to be reflected in the calculation of recall values. The ASJC-based subject sizes had to be deflated to derive estimates that could be compared with ADS data (a value that counts each record once), so each subject was calculated based on fractional counting (Perianes-Rodriguez et al., 2016), a value referred to here as single-attribution. To calculate the number of single-attributed subject sizes in Scopus, I needed to determine the *specific* level of multi-attribution of each of the 26 subjects. Using the average of 1.59 subjects across all records would over- and under-value single subjects. First, I determined the number of records that were only attributed to a single subject and then calculated the fraction of records assigned to two or more subjects. The specific multi-attribution factors showed that records in Medicine, for example, are attributed to 1.38 categories on average. In contrast, Decision Science’s and Material Science’s multi-attribution factors were as high as 2.75 and 2.67, respectively. Knowing the *subject-specific* multi-attribution factors, I could determine the recall of a keyword for a specific subject, whereas 100% recall would amount to the sum of records on a database and not the sum of records assigned to subject categories.

### Step 2: collection of estimates and calculation of the aggregate estimate

Harvesting individual estimates of subject coverage required using the most restrictive field code to retrieve the QHCs for the 364 keywords (26 subjects × 14 keywords) for each of the 56 databases. Each QHC value divided by the subject-specific recall unique to each keyword (as determined in Step 1) was matched with the appropriate field code data. For example, if QHCs were collected via the ‘title’ field codes, the recall values for the ‘title’ field codes were also used. Generally, ‘title’ data was preferred, as the WOS reference data showed that ‘title’ estimates were the most accurate. Parentheses and other symbols were used to limit the search results to the verbatim meaning of the keywords and to exclude automatic stemming or query expansion, wherever possible. Queries were kept as simple as possible, for example, by using the same field codes and unigram keywords, to maximize compatibility of the approach over a maximum number of databases. Accordingly, no filters were applied nor were any other steps taken to manipulate the query results. The resulting values were independent estimates of subject coverage of the 56 databases.

#### Calculation of aggregate estimates at different precision levels

Individual estimates with a precision measurement above a specific threshold were used to calculate the ‘aggregated estimate’—an estimate with higher average accuracy than individual estimates. The ‘aggregated estimate’ accounted for differing accuracy levels, outliers, and other artifacts and excluded estimates below a precision threshold from the calculation. The exclusion of low-precision estimates was important as low-precision estimates were found to systematically produce inaccurate estimates. Using the WOS control dataset, I could determine the accuracy of the estimates at various precision levels. The result showed that the accuracy of the subject estimations was lowest at very low and very high precision thresholds. The lower the precision threshold, the less precise the estimate as too many outlier values were included. Outliers are matches not due to the intended meaning of the keyword. An example of very low precision would be the keyword “comedy” estimating the subject coverage of Engineering. The results of such estimation are far off the actual value. The WOS control dataset showed that determining the precision threshold of keywords was important for the overall accuracy of the estimations.

The theoretical maximum number of estimations in the dataset would be 9464 if estimates were available for all 364 keywords for all 26 subjects. However, the number of estimates falls considerably with more restrictive precision thresholds, excluding the artifacts low-precision keywords produce. Precision thresholds that are too high would exclude many keywords that do not meet the threshold. The effect would be to reduce the number of estimations per calculation, thus also increasing the likelihood of artifacts. The use of the ‘title’ field code at the 17% level encapsulated 660 estimations. At the 30% level, that number falls to 380. Some subjects have less precise keywords, which renders analysis more difficult. Specifically, Decision Sciences, Health Sciences, and Chemical Engineering have the least discriminant language, while Earth and Planetary Sciences, Medicine, and Physics have highly-specialized keywords.

For the ‘abstract’ field code and the ‘all fields’ field code, the number of estimates is slightly lower at the respective precision thresholds, as the field codes include more sections of document text and thus are less precise. Accordingly, to include as many QSCs as possible at a given precision level, the most restrictive field code ‘title’ was chosen for the analysis whenever the search system supported the field code.

#### The median as a robust aggregate estimate based on many QHCs

A robust model based on the median was chosen to ensure the accuracy of subject estimation. Robustness was achieved by basing the estimation on a large set of keywords that produced a large number of independent estimates. The reasoning was, if a system had, for example, high coverage in Medicine, it would not only cover a significant share of records for ‘pain’ but also for other medical keywords. However, a system *only* having high coverage of ‘pain’ but not of other keywords from Medicine could be because of at least one of the following reasons: First, the QHC is an artifact as the keyword is used in a notably different way on this database than in the Scopus dataset (e.g., ‘pain’ is interpreted as a name rather than a medical symptom). Second, if the keyword was processed differently to the reference dataset (e.g., a query that is assumed to be interpreted as verbatim was in fact expanded). Third, the QHC is a correct estimate, yet the database does not cover (much) more from the subject. In all these cases, the median ensures that the overall subject coverage is not inflated by extreme or outlier values but reflects the estimate for the coverage of an entire subject, as determined by ASJC. Accordingly, the median was used to calculate the ‘aggregate estimate’ at different precision levels.

#### Median of medians, a robust, parsimonious model for all databases

Analyzing the WOS control dataset shows that the most optimal precision threshold for the aggregated estimates differs across different field codes. Similar differences were found when using verbatim vs. stemmed queries. Based on these differences in optimal precision thresholds, I reasoned that no single precision level was optimal for the most accurate estimates. Accordingly, the optimal precision threshold most likely also varies between databases.

I decided that generating a robust model would be more likely if, instead of focusing on a *single* precision threshold level, I sought to balance accuracy (reasonably precise estimates) and robustness (many estimates) by using a range of precision thresholds. The WOS control dataset showed the broadest array of acceptable precision thresholds was 17–30%. Precision levels above 30% increasingly included ever more missing values while levels below 17% included more artifacts. Calculating the aggregation of the estimates within this range again relies on the median of precision levels. This method again reduces the effect of outliers from the array of values between 17 and 30% precision. This method is termed the ‘median of medians’ in mathematics and is applied in deterministic sorting and in selecting algorithms (Sen & Kumar, 2019). Using a median across precision levels ensured the reduction of outliers when specific precision thresholds produced significantly different results or artifacts. The model produced aggregate estimates of all 26 subject categories following these steps.

#### Methods that did not improve overall accuracy

In determining the parsimonious model chosen in this study, I also assessed alternative methods to that described above. Those were either disregarded due to the high likelihood of overfitting or lower levels of accuracy. It was important to use the median within a specific threshold range (17–30%), as the median over *all* estimations or *all estimations above a threshold* produced weaker results. In contrast, all *mean*-based calculations were neglected because subject-specific databases in particular produce more outliers. What also led to inferior results was *precision weighting* and *precision weighting normalized by relative subject-precision levels*. While the precision levels were important for the optimal model, *recall* was found to be an inaccurate model calibrator. As these models did not yield significant improvements that would be robust across all databases, I chose the most parsimonious and conservative model to limit the issue of overfitting the model to the WOS CC control dataset.

### Step 3: normalization of aggregate estimates (sum is equal to the number of records)

To improve accuracy, it was necessary to normalize the values of the aggregate estimates of the absolute number of subject-specific records on a database derived from step 2. Normalization accounts for this systematic issue of under- or overestimations of absolute subject coverage. Deviations might occur due to systematic differences in the scope of field codes; for example, when the reference database uses the ‘abstract’ field code and the database under investigation uses both the ‘title’ and ‘abstract’ field codes (in case of Dimensions). Then, because of a systematically wider scope of the QHCs, the aggregate estimates are likely to overestimate *absolute* subject coverage. While the *relative* shares of subjects likely stayed the same, normalization ensured that the estimates were properly scale-adjusted.

Normalization was undertaken via the information on absolute database size, determined by the number of scholarly records available on a database. The ADS information was collected from several sources: If the ADS information was published on an ongoing basis by the database provider and was up to date, the normalization relied on that information. However, many databases do not publish that information or only provide outdated ADS data. In that case, I had to rely on the proven method of QHC data (Gusenbauer, 2019), where a query retrieves the entire dataset available on a database. Initially, the use of ‘absurd queries’ to determine database sizes was pioneered by Orduña-Malea et al. (2014), a method having *“the advantage of not being affected by estimates taken from biased databases” (*Orduña-Malea et al., 2015*, p. 945).* If the QHC method was not working, I relied on the latest, albeit outdated, information on database size from the database provider as the next best estimate. After normalization, possible systematic under- or overestimations were accounted for, and the resulting absolute and relative subject coverage reflected the best estimates of the BOK method. To illustrate the rationale of these steps, an example estimation based on the chosen BOK model is shown in Table 3.

**Table 3:** Estimation of Subject Coverage of Agricultural and Biological Sciences at WOS CC (via QHCs based on title queries; selected WOS CC indexes see Table 2)

Step 2	Estimates based on precision thresholds between 17 and 30%, determined by median over the QHC-based estimates	Precision threshold	Number of QHCs	Estimates based on median
		Above 17%	26	5,269,192
		Above 18%	25	5,250,991
		Above 19%	25	5,250,991
		Above 20%	22	5,038,115
		Above 21%	22	5,038,115
		Above 22%	20	4,779,398
		Above 23%	18	4,620,765
		Above 24%	18	4,620,765
		Above 25%	17	4,556,258
		Above 26%	17	4,556,258
		Above 27%	17	4,556,258
		Above 28%	17	4,556,258
		Above 29%	16	4,457,038
		Above 30%	16	4,457,038
	Aggregated estimate determined via median of medians (17–30%)			4,620,765 (sum of all subjects: 117,385,940)
Step 3	Normalization of estimate via the absolute size of WOS CC			3,137,832 (sum of all subjects: 79,713,501, equals ADS)

## Results: estimates of absolute and relative subject coverage

The estimates of subject coverage were determined to answer a central question: *“where am I likely to find the most relevant records for my specific search goal?”* Two search goals can be distinguished: the exhaustive strategy (high recall) and the context-focused strategy (high precision). I will present the results ranked so as to inform those strategies to allow optimal database selection. First, results are ranked by absolute coverage of each subject (recall). Second, they are ranked by relative coverage of each subject (precision). Additionally, the results sorted by ADS are available in the “Appendix”. Due to the great wealth of data, the results section only describes some noteworthy general findings. For many readers the most helpful tables will be those describing the greatest *absolute* and *relative* coverage of databases in their specific subject. How those results might be applied will be described in the discussion section.

### Database estimates ranked by absolute coverage of each subject (level of recall)

**Fig. 1:**
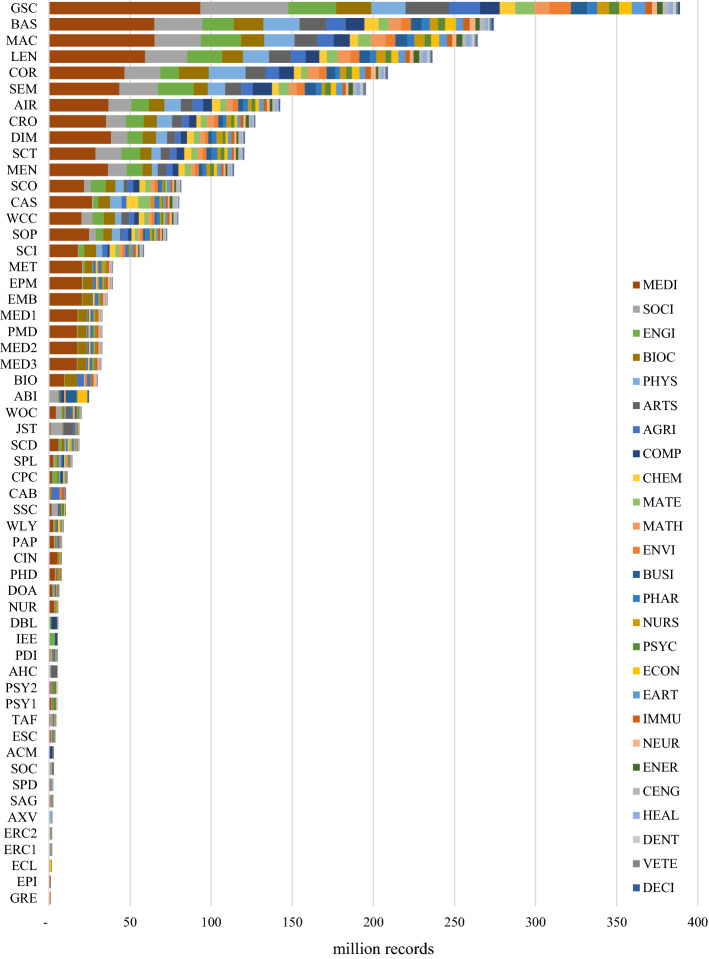
Absolute subject coverage of 56 databases, sorted from largest to smallest (based on single-attribution; abbreviations see last page of Table 2)

Researchers wishing to search among the greatest number of relevant records need to choose a database with the highest absolute coverage of their chosen subject(s). Table 4 also depicts the absolute size differences of the individual subjects, while Fig. 1 illustrates the magnitude of size differences of individual databases and the subjects they cover.

#### Most comprehensive coverage and runners-up

In 19 of the 26 subjects, Google Scholar has the greatest coverage. It is estimated to be the dominant player and to have an unrivaled ability to amass a unique quantity of scholarly records through a combination of publisher cooperation and crawlers. Microsoft Academic covers most in Energy, Dentistry, and the Health Professions. BASE has the most coverage in Decision Sciences and Neuroscience; Core in Physics and Astronomy, and Semantic Scholar in Veterinary. The remaining subjects are all covered to the largest extent by Google Scholar.

For some subjects, the largest database covers substantially more than the second-largest. This difference is greater than 30% in Environmental Science (53%), Social Sciences (45%) and Earth and Planetary Sciences (42%), Arts and Humanities (39%), Agricultural and Biological Sciences (37%), Materials Science (32%), Psychology (31%), and Medicine (30%). In all these subjects, Google Scholar provides substantially more coverage than alternatives, which underlines the great significance of the search engine. These findings are in line with previous research examining the overlap between six large search systems (Martín-Martín et al., 2021). Among the databases analyzed, Google Scholar was found to have the greatest coverage and the largest share of unique records in all of the eight subject areas used by the authors. Compared to this study, BOK estimates identified two interesting findings: First, while Martín-Martín et al. (2021) show Google Scholar has superior coverage in all of the eight categories analyzed, BOK shows it does not have superior coverage in all of them (analyzing a larger sample of databases). Second, their analysis of Google Scholar’s share of unique records can largely also be seen in the data of the BOK estimates. Martín-Martín et al. (2021) found that Google Scholar covers many records that are not found among the other five databases; particularly in ‘Humanities, Literature & Arts’ (53%), ‘Business, Economics & Management’ (47%), ‘Social Sciences’ (45%). In this study, both Social Sciences (45%) and Arts and Humanities (39%) are among the subjects where Google Scholar surpasses all other databases by far. In the third subject, Business, Economics & Management that Martín-Martín et al. (2021) identified as rather uniquely covered by Google Scholar, BOK estimates show a smaller difference. This is mainly because of BASE’s substantial coverage of Economics, a database not analyzed by Martín-Martín et al. (2021).

#### Subject sizes

The data show that Medicine, with 93 million records, is by far the largest discipline, according to Google Scholar estimates. Second and third are Social Science and Engineering with 54 million and 30 million records each. The smallest subjects are Veterinary (1.3 million), Decision Sciences (1.3 million), and Dentistry (1.9 million).

#### Databases and their promises

The BOK data also show that across subjects, large, multidisciplinary databases *always* cover more than the specialized databases in the sample. The closest a specialized database came to covering most records was ABI/Inform Global in Economics, Econometrics, and Finance. It covers 5.9 million records from this subject, while Google Scholar only covers 1.8 million more. This relatively small difference is because ABI/Inform Global contains many trade publications and newspaper articles in addition to journal articles and other scholarly content. These records are mostly not covered by Google Scholar.

The comparable low absolute coverage is noteworthy also because of the many database providers that claim ‘most comprehensive’ coverage in their fields; claims that have not been verified until now. Within the dataset, BIOSIS Citation Index, SocINDEX, SPORTDiscus, and ACM Guide to Computing Literature claim superior comprehensive coverage. BOK estimates indicate that these claims are clearly not substantiated. The results show that while those databases are important, and perhaps essential in their fields, superior coverage is provided by the large, multidisciplinary databases. What these databases do particularly well is, however, high *relative* subject coverage, that is high specialization in individual subjects. These results are presented next.

**Table 4:**
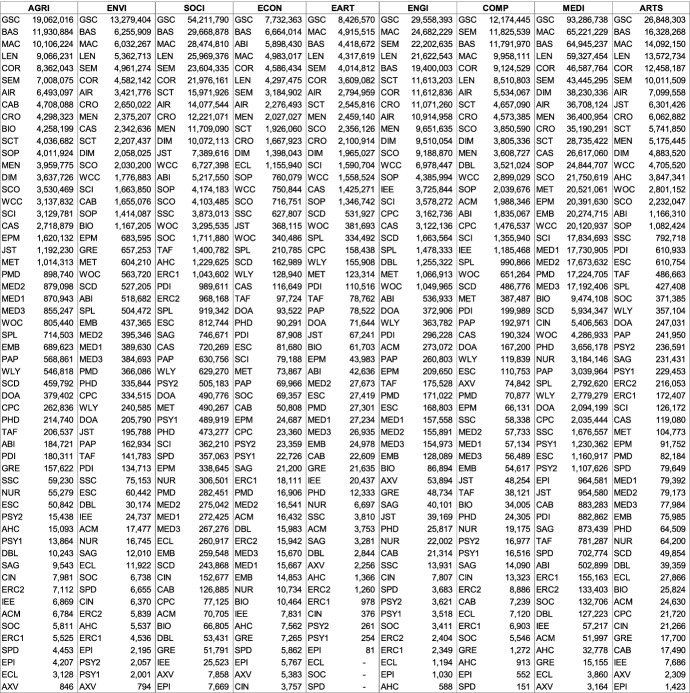

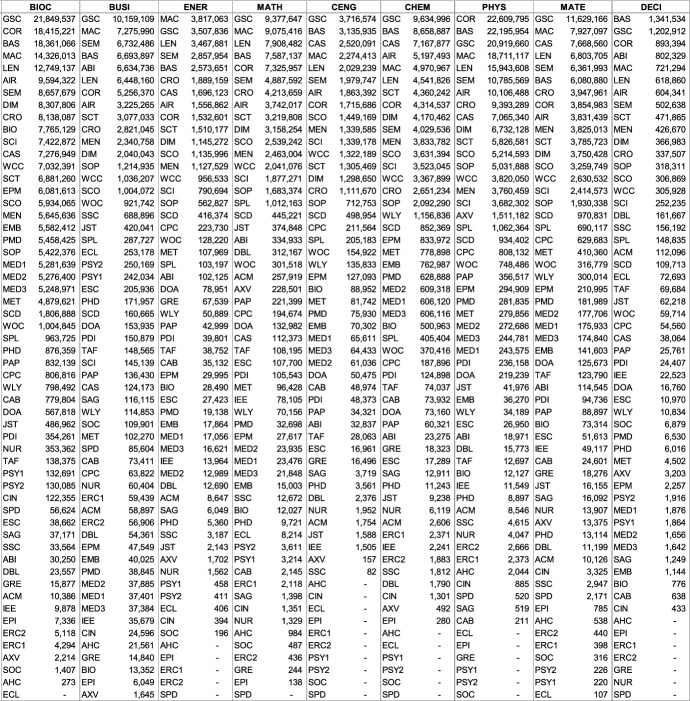

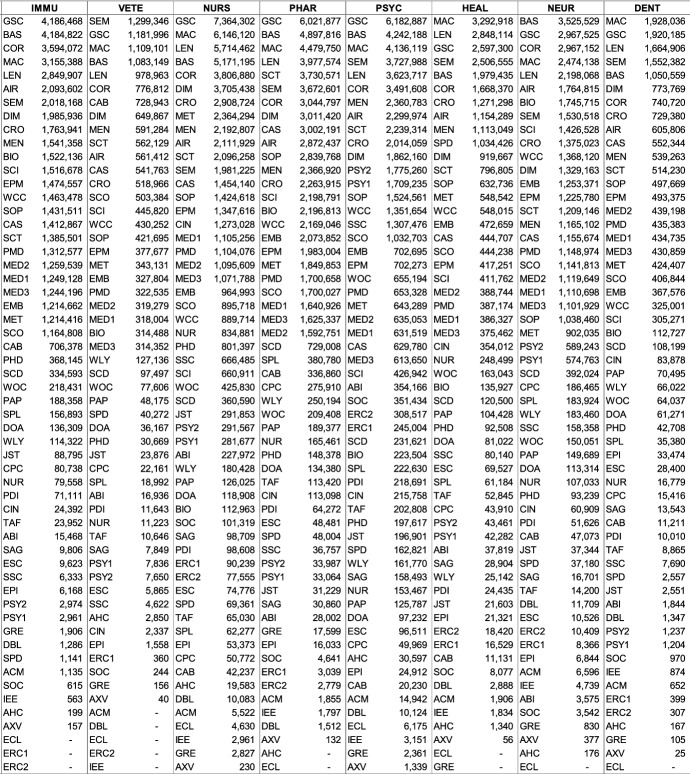
Absolute coverage ranked for each subject (based on single-attribution; abbreviations see last page of Table 2)

### Databases estimates ranked by relative coverage of each subject (level of precision)

**Fig. 2:**
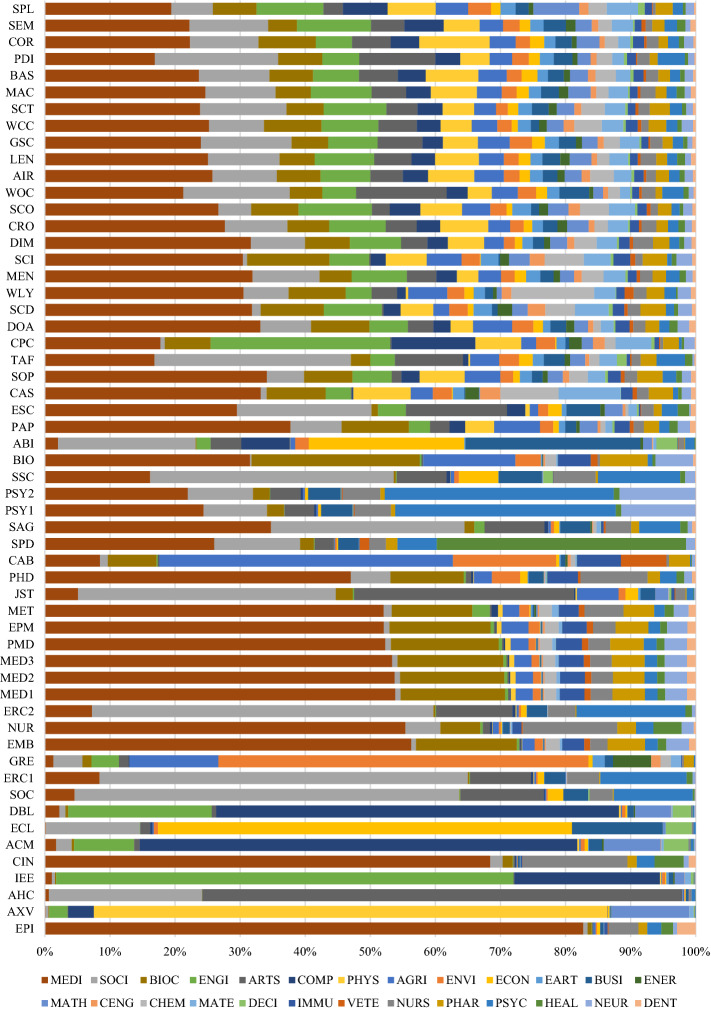
Relative subject coverage of 56 databases, ranked from least to most specialized (based on single-attribution; abbreviations see last page of Table 2)

Choosing the database with the highest *absolute* coverage is not always ideal. If it were, researchers would likely be searching with Google Scholar in most situations. For search goals targeting precise results, it makes sense to search through databases with high *relative* subject coverage (see ranking in Table 5 and an overview in Fig. 2). Other considerations besides absolute and relative coverage, necessary to select the optimal database, are described in the discussion section.

#### Level of specialization

The variance of relative subject coverage was calculated to determine a database’s level of specialization. Low variance indicates subjects are evenly distributed and that the database is multidisciplinary. High variance indicates they are unevenly distributed and the database is specialized. The first four columns of Table 5 show a ranking of the level of specialization of the databases in the sample and information on its openness. A comprehensive overview of relative subject coverage across all 56 databases is illustrated in Fig. 2. The least specialized database, with the most even coverage across subjects, is SpringerLink, followed by Semantic Scholar and Core. It is noteworthy that BOK estimates could substantiate the claim by Springer Nature (2022) of SpringerLink being “… *the world’s broadest multidisciplinary collection of online resources covering life, health, physical, and social sciences and the humanities*.” Among the 56 databases analyzed, SpringerLink is indeed the most multidisciplinary by a significant margin. The most specialized database, with the most diverse coverage across subjects, is Epistemonikos, followed by arXiv and the Arts and Humanities Index. Assuming a (somewhat arbitrary) threshold value of 50%, a total of 25 databases can be considered multidisciplinary. The remaining 31 databases are considered specialized.

All large databases that cover around or more than 100 million records are multidisciplinary. Nevertheless, not all multidisciplinary databases are large: SpringerLink, ProQuest Dissertations & Theses Global, WorldCat-Thesis/dissertation, Wiley, ScienceDirect, DOAJ, the Conference Proceedings Citation Index-Science, Taylor and Francis, and the Emerging Sources Citation Index all cover less than 25 million records and are considered multidisciplinary.

#### The ‘new players’ versus the established (Google Scholar, WOS CC, Scopus)

Among the 56 selected databases are several recently relaunched new players: Microsoft Academic, Dimensions, scite, Lens, Semantic Scholar, or Meta. In terms of absolute subject coverage, only the databases underlying Semantic Scholar and Microsoft Academic can outperform Google Scholar, and then in only a few subjects. Overall, Google Scholar still is by far the most comprehensive resource across almost all subjects. With the discontinuation of Microsoft Academic in 2021, academic discovery has lost its runner-up in many subjects.

How do the remaining new players compare in terms of relative coverage? Dimensions, scite, Lens, Semantic Scholar are multidisciplinary databases with large areas of similar subject focuses; however, for specific subjects, they differ. For example, Semantic Scholar focuses more on Engineering, while Dimensions focuses more on Medicine and less on Social Sciences. WOS CC, as the go-to solution for many researchers, has a different subject focus than the new players. WOS CC features the Arts and Humanities Index and the Social Science Citation Index leading to a relatively greater focus on these subjects. The difference is considerable. While ‘the new players’ have a 4–6% focus on Arts and Humanities, WOS CC has 14%. Similarly, WOS CC covers 16% Social Science, while the new players cover 8–13%. For Engineering, the reverse is true: WOS CC covers 5% while the new players cover 8–11%. However, it is important to note that the WOS CC can contain up to seven different indices (which are subdivided into multiple versions). The number of indices the WOS CC contains and those indices’ retrospective coverage will differ among subscriptions between institutions. An advantage of WOS CC is that it allows the de-selection of single indices to permit researchers to customize their search scope. In addition to analyzing the WOS CC as a set of indices, this study also analyzed six^3^ of the indices WOS CC can cover individually. That information can help researchers know which indices to include or exclude when searching using the WOS CC.

Interestingly, Scopus has a considerably lower coverage of Social Sciences and greater coverage of Engineering than all the other databases compared in this section. Scopus’ greater focus on the Physical Sciences, in general, is also visible in its greater coverage of most of the subjects summarized by this subject area (see Table 1). These differences in subject coverage between databases underline the importance of investigating absolute and relative subject coverage of both the new and established players when designing an optimal search strategy. WOS CC and Scopus are not as similar as they seem in all subjects. This is a new finding, as previous studies have found that Scopus and WOS CC overlap considerably (Martín-Martín et al., 2018b, 2021). The divergence in findings might be accounted for by the different methodologies used or differences in the underlying WOS CC versions. I will discuss this in more detail in the discussion section.

The new player not yet discussed is Meta, a database that is in a different game, as it specializes with a deliberate focus on Health Sciences. Compared to the established databases of PubMed, Europe PMC, Medline, CINAHL Plus, or Embase it would have provided a viable alternative but is set to be discontinued in early 2022. While Meta is largely similar in relative subject coverage, it is noteworthy that Meta seems to cover a larger share of Nursing records. Comparing these databases reveals how CINAHL Plus has a significantly greater focus on Medicine, Nursing, and Health Professions than the other databases; a finding that might not be obvious from textual descriptions. Conversely, with only 2% CINAHL Plus has a much smaller focus on Biochemistry, Genetics and Molecular Biology, while the others have 12–17%.

It is important to remember, the new players are not only interesting in terms of their subject coverage but also because of their unique approaches to knowledge discovery. This emphasis can manifest in terms of advanced filtering, data handling, innovative citation information, or ranking metrics.

#### Subject focus of specialization

It is noteworthy that the relative coverage of subjects in the dataset is very diverse. There are few subjects with high relative coverage, while there are many with low relative coverage. The great focus of scholars on Medicine is visible in the great relative coverage of the subject among most of the 56 databases as 29 databases cover at least 25% and ten cover more than 50% in Medicine. Epistemonikos contains most Medicine topics with 82.7% coverage. After Medicine, the second most covered subject is Social Sciences, a fact also noticeable in the high absolute coverage of the subject.

For nine of the 26 subjects, no database covers more than 10% of the subject. This lack of subject focus is due to two reasons: first, some databases specializing in these subjects were not included in the analysis, and second, there is some subject overlap where ASJC assumes most coverage is attributable to other subjects. In the cases of the Nursing & Allied Health Database and the Public Health Database: both are described as focusing on Nursing, yet their greatest relative coverage is Medicine, presumably because Nursing, Medicine, and other health-related subjects overlap, which causes BOK to determine that their focus lies on Medicine rather than Nursing. However, this discrepancy does not impede selecting the most specialized Nursing databases, as the BOK weighting is the same across all databases in the comparison. The comparison shows that the best database for Nursing is CINAHL Plus with 16% coverage, followed by the Nursing & Allied Health Database and the Public Health Database.

#### Open access?

For researchers without institutional access, searching specialized databases is not as easy as searching multidisciplinary ones. Of the 56 databases analyzed, 30 are openly searchable. Of these 30, 60% are multidisciplinary, showing that most specialized databases in the sample are behind paywalls maintained by aggregators (Web of Science, Ovid, ProQuest, EBSCOhost). Nevertheless, searchers selecting one of the openly accessible systems will however not find that open discovery—the search and access of scholarly content via freely available resources—is possible. The data show that the open-access rates of the records available on openly accessible databases differ substantially (see Tables 2 and 5). Particularly journal platforms of the large publishers still have open access rates in single-digit percentages, which means that although their content is searchable, almost all of it is behind a paywall. Accordingly, open discovery is still very limited, both when searchers want to access multidisciplinary or specialized databases.

**Table 5:**
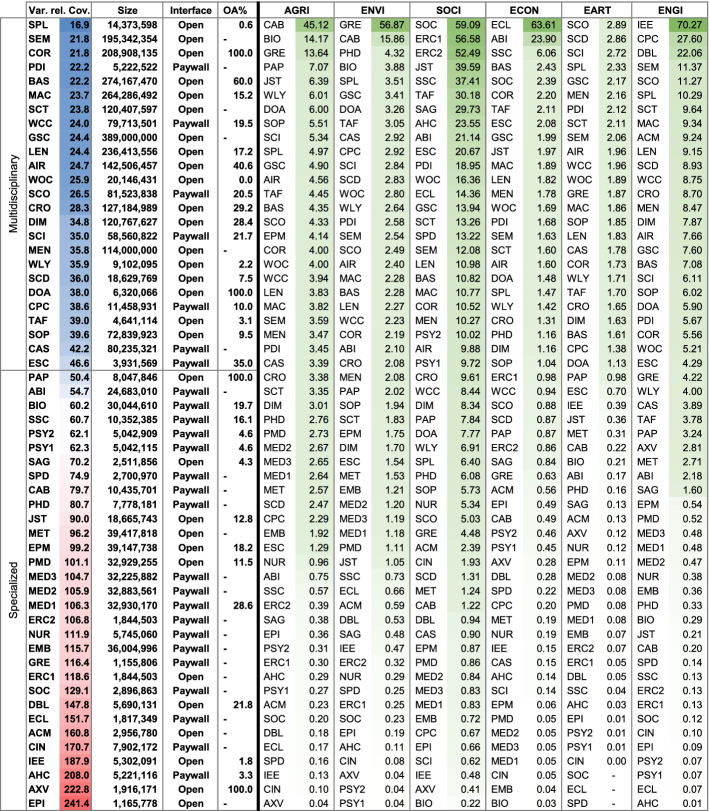

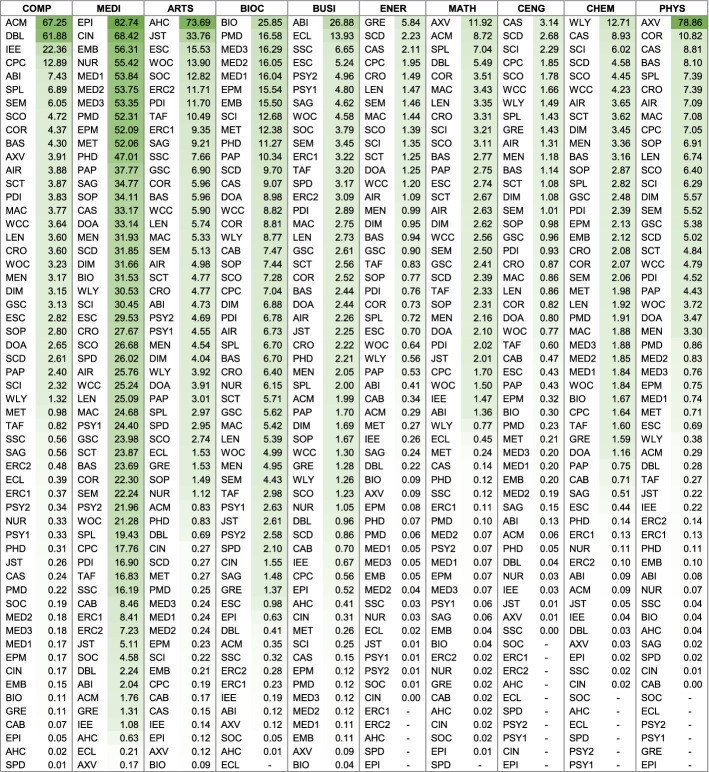

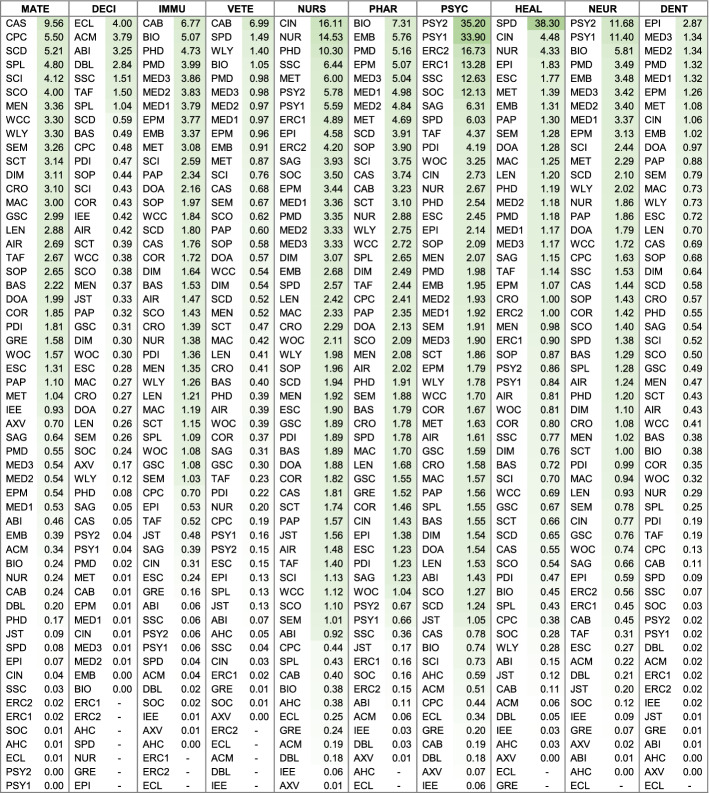
Relative coverage ranked for each subject (based on single-attribution; abbreviations see last page of Table 2)

## Validation of results

It is important to scrutinize the results of any research claiming to guide search decisions. I conducted several tests to determine the validity of the BOK estimation method (internal validity) and the accuracy of the results (external validity).

### Internal validity: are estimates comparable between each other?

As the method of determining subject coverage estimates is the same, or very similar, across systems, the *comparability* across systems generally can be rated high. A mechanism that generally assures high internal validity of the BOK method is if a system has biases in determining the QHC (e.g., stemmed queries or automatic keyword expansion), it is likely to have the same biases across all subjects. Accordingly, while the median sorts out single outliers, any systematic biases the system has over most or all estimates are likely to be netted out because the method calculates relative shares of a subject category across the total. As the ADS is known for all databases, the relative shares can be used to determine the best estimate of the absolute coverages of subjects. To assess internal validity, I compared the estimates across systems and variations of the BOK method.

#### Same databases, estimated via different systems

Comparing the same databases across different systems (e.g., Medline via PubMed, Ovid^4^, Web of Science, and EBSCOhost) shows that the method produces very similar results across systems. The Pearson correlation coefficient of the subject coverage for PubMed versus Medline (via WOS) and PubMed versus Embase (via Ovid) is 0.998 and 1.000, respectively. Among the Medline databases (including PubMed) absolute coverage rates are broadly similar, and the small differences are mostly attributable to the different updating speeds of database providers (i.e., EBSCOhost, WOS, Ovid). The absolute sizes of the databases vary between PubMed’s and Medline’s 46 million and Embase’s 50 million records (multi-attribution estimates), while the relative coverage rates are similar. This shows BOK’s ability to estimate consistent results: the system detects similar databases accurately.

#### Relative interquartile range (RIQR) as an indicator of the consistency of estimates

The *relative interquartile range (RIQR)* of estimates was the indicator used to show how uniformly single QHC values estimated the coverage of a specific subject. RIQR is used to indicate the robustness of the median against outliers. It is a value that indicates the level of homogeneity of the underlying estimates. (Johnson & Bhattacharyya, 2010). RIQR is calculated by dividing the IQR by the median to obtain a relative value comparable at different rates of absolute coverage. The data show that RIQRs are particularly high for subjects with low levels of coverage. This effect may be explained by a small median value making RIQRs appear artificially high at low coverage subjects. In addition, in the case of low-coverage subjects, some keywords produce zero or very low QHC rates, while other keywords attributable to the same subject might produce significant QHC rates owing to the presence of artifacts. If there is almost no coverage of a subject, then word variations, glitches, ambiguities come into play far more than with higher-coverage subjects. Because of these discrepancies between QHCs, the RIQR can be high.

To detect high RIQRs beyond low-coverage subjects, I also calculated RIQRs for all subjects with at least 2.5% coverage (RIQR2.5). Among all 56 databases in the analysis, 19 had a mean RIQR2.5 of above one, meaning that the interquartile range (Q3–Q1) was more than its median, even for subjects with higher than 2.5% coverage (for data, see “Appendix”). With values clearly above 2, the highest RIQR2.5 rates were prevalent in the Arts and Humanities Index (4.0), ERIC (3.2 and 2.75), SPORTDiscus (2.34). All of those databases have a narrower focus than the categories of ASJC (ERIC or SPORTDiscus) or cover subjects that are inherently very diverse, like Arts and Humanities (Arts and Humanities Index). In all of these cases, high RIQR2.5 rates are plausible and show the limitations of the method when the subject focus of systems is too narrow. Then the selected keywords are less representative of the overall subject. The data show that RIQR is a useful tool to assess the validity of the estimates at the database level. As the results of the coverage of the databases were plausible, they were not excluded from the results.

#### Different field codes

Not all systems allow searching their databases with the preferred ‘title’ field code. Therefore, it was necessary to additionally use the ‘abstract’ and ‘all fields’ field codes for data collection with some databases. For the WOS control dataset, estimations for all three field codes were extracted. The data showed that ‘title’ estimates were the most accurate. The accuracy (mean deviation) of the estimates was 19.6% for ‘title’ searches, 21.2% for ‘abstract’ searches, and 22.3% for ‘all fields’ searches. The estimates for the 26 subjects correlated between ‘title’ and ‘abstract’ at 0.980 and between ‘title’ and ‘all fields’ at 0.985. Accordingly, while the ‘title’ field code is preferable, other field codes work reasonably accurately to estimate subject coverage.

#### Verbatim versus expanded queries

The goal of the BOK method is to ensure comparability across systems, and accordingly, the process was designed to keep as many aspects as constant as possible. The ideal estimation method would use verbatim queries based on exact matching. Estimations based on stemmed matches were validated because not all systems supported exact match estimations. Stemmed queries were tested using QHCs and QSCs based on stemmed data for both WOS and Scopus. The results show a Pearson correlation between the verbatim and stemmed estimates of 0.999. While this seems reasonable, the accuracy of the estimate decreased from 0.196 to 0.224 and the RIQR increased only slightly from 0.31 to 0.36. The effect showed that while QHCs are relatively consistent (low RIQR), estimates are likely to be less accurate than with more restricted queries.

### External validity: Are estimates accurate so that they actually reflect subject coverage?

The external validity of the BOK estimates was assessed by determining their accuracy in reflecting actual subject coverage of a database. As RIQRs showed differences between multidisciplinary and specialized databases, I have assessed external validity for these groups of databases separately: Multi-disciplinarity is assumed with a variance of relative subject coverage below 50%, specialization is assumed with variance above 50%. Further, the databases of ERIC and SPORTDiscus, which both have a very narrow subject focus, were analyzed separately.

#### Multidisciplinary databases

I used the *WOS control dataset* to determine the accuracy of multidisciplinary systems. WOS CC uses the WOS classification system consisting of 254 categories at its most granular level in 2021. WOS classification, like ASJC, is considered relatively robust as it uses a manually curated classification mechanism. These two classification systems are probably the best candidates for pairwise subject comparison.

When comparing both classification systems, I first matched and grouped the 254 WOS categories to the 26 ASJC categories. I then determined the subject coverage of WOS CC via QHC of the grouped WOS categories. This process ensured the QHC data indicated the number of records in WOS CC in terms of ASJC classification. The systemic differences in the extent of multi-attribution between both systems (Wang & Waltman, 2016) meant I had to make the QHC results of both classification systems comparable. Accordingly, I deflated the multi-attribution of both datasets, so that both sums of subject attributions matched the number of records on a database. As a result, I could compare WOS subject data retrieved via exact subject queries translated into ASJC classification with the BOK estimate based on QHCs.

I followed the same logic to create different control datasets to determine the accuracy of variations of the QHC method. I tested different field codes, verbatim/stemmed versions of keywords, precision levels, and variations in the estimation method (see Step 2 and Step 3). That process helped me understand the workings of the BOK and QHC method in general. Overall, it was important not to fit the model to the database but to allow the most accurate estimates across databases. Accordingly, the simplest method that is likely to be reliable across databases was chosen as the standard for all databases. The method used in this study (i.e., most restrictive field code and restrictive, verbatim queries) produced a mean accuracy of the WOS estimate of ± 19.6% with a maximum deviation of 46.6% (see the comparison in Table 6).

While this direct comparison between WOS and ASJC classification was very helpful in determining variations of the BOK method, the absolute accuracy data should be treated with caution. Perfect matching of both classification standards is unlikely to be possible at the aggregate level due to both systems extracting different boundaries in assigning categories to records. For example, ASJC would classify records from the WOS subject ‘OPERATIONS RESEARCH & MANAGEMENT SCIENCE’ partially to Decision Sciences and partially to Business, Management and Accounting. Such inaccuracies cannot be accounted for when matching categories. The alignment of both multi-attribution standards is another source of potential inaccuracy. Given these limitations, the comparative results using the control dataset cannot be interpreted as being precise. Nevertheless, the process can illuminate how various calibrations of the BOK model affect the accuracies of precision levels. The WOS comparison dataset proved helpful in assessing the quality of BOK and QHC method and the performance of method variation choices. The estimated mean accuracy of ± 19.6% gives an impression of the level of accuracy that can be expected. Consequently, the external validity of the BOK method can be rated high for multidisciplinary databases.

**Table 6:** External validity of Subject Coverage Of Agricultural And Biological Sciences At WOS CC (via QHCs based on title queries; selected WOS CC indexes see Table 2)

Step 3:	Normalization of estimate via the absolute size of WOS CC based on single-attribution		3,137,832 (sum of all subjects: 79,713,501)
External validation	Estimated WOS CC coverage based single-attribution		3,049,334 (sum of all subjects: 79,713,501)
	Relative accuracy of the aggregated estimate (the mean deviation for WOS CC over all subjects is 19.6% or a total of 15,176,745 records, with a range of − 46.6% to + 45.5%)		deviation of aggregated estimate: + 2.9%

#### Specialized databases

Specialized databases have high coverage rates of a few subjects and low coverage of most other subjects. That characteristic is reflected in a high variance in relative subject coverage. To verify external validity, I looked at the plausibility of BOK estimates for the six most specialized databases in the dataset: Epistemonikos with the greatest variance in relative coverage (241.4%), arXiv with the second greatest (222.8%), Arts & Humanities Citation Index with the third greatest (208.0%), IEEE Xplore with the fourth greatest (187.9%), CINAHL Plus with the fifth greatest (170.7%) and ACM Guide to Computing Literature with the sixth greatest variance (160.83%). For these specialized databases, I verified the external validity of results by referring to textual descriptions of the dataset (see Table 2).

Epistemonikos’ (2021a) self-description states: *“Epistemonikos is a collaborative, multilingual database of health evidence. It is the largest source of systematic reviews relevant for health-decision making, and a large source of other types of scientific evidence.”* According to the BOK estimates, it is the most specialized database and with 83% covers by far the greatest share of records in Medicine. The runner-up is also highly specialized, CINAHL with 68%. In addition to Medicine, the Epistemonikos content is some 5% Nursing, 3% Dentistry, 2% Psychology 2% Health Professions, and 1% Pharmacology, Toxicology and Pharmaceutics. The remaining 20 subjects cover well below 1% and some even have zero coverage, according to BOK. Epistemonikos (2021b) states it excludes records from coverage if they *“do not address a health problem (we do not use an explicit definition of health, but encourage our collaborators to understand health in a broad sense), [or if they] …do not evaluate individuals or groups of individuals (except for some topics that can inform health decision-making, for instance bacterial resistance to antibiotics, levels of environmental chemicals in food).”* Accordingly, Epistemonikos can be seen as a rigorously curated, highly-specialized dataset on health decision-making. This great level of specialization is well reflected by the BOK estimates, which can be rated highly plausible.

arXiv’s description indicates it covers the following subjects: *Physics, Mathematics, Computer Science, Quantitative Biology, Quantitative Finance, Statistics, Electrical Engineering And Systems Science, And Economics*. This textual statement tells the researcher little about the specific weighting of these subjects. According to the BOK method, arXiv covers 79% Physics and Astronomy, 12% Mathematics, 4% Computer Science, 3% Engineering, and 0.3% Economics, Econometrics and Finance—a substantially clearer picture on subject coverage than provided by the textual description.

Clarivate Analytics’s (2021) description of the Arts & Humanities Citation Index states: *“Arts & Humanities Citation Index contains over 1800 journals across 28 arts & humanities disciplines.…Our expert in-house editors use a single set of 28 criteria throughout the journal selection and curation process.”* BOK rated the database as highly specialized and estimated its subject coverage at 74% Arts and Humanities and 24% Social Sciences. All other categories are estimated well below 1%, 17 subjects cover 0% or close to 0%.

IEEE (2021) states it *“provides web access to…publications in electrical engineering, computer science, and electronics.”* The IEEE Xplore categories ‘electrical engineering’ and ‘electronics’ can probably be associated with the ASJC category ‘Electrical and Electronic Engineering’ which is a sub-category of ‘Engineering’. BOK estimates IEEE Xplore’s subject coverage at 70% Engineering, 22% Computer Science, 1% Mathematics, 1% Medicine, and 1% Materials Sciences. The remaining subjects are covered to an even lesser extent, reflecting the high degree of specialization of the database. With BOK, the written statement is quantified and informs that Engineering is more than three times more prevalent than Computer Science.

EBSCOhost (2021b) describes that CINAHL Plus *“indexes top nursing and allied health literature.”* The BOK method indicates it covers 68% Medicine, 16% Nursing, 4% Health Professions, 3% Psychology, 2% Social Sciences, 2% Biochemistry, Genetics and Molecular Biology, 1% Dentistry. Among all databases in the dataset, it is the top Medicine and Nursing database (except for Epistemonikos that focuses on evidence syntheses) with the highest relative coverage in both subjects. After SPORTDiscus which is focused on sports health, CINAHL Plus is the second most specialized database in Health Professions.

The Association for Computing Machinery (2022) describes the Guide to Computing Literature as *“the most comprehensive bibliographic database in existence today focused exclusively on the field of computing.”* If comprehensiveness means *relative* coverage, then BOK confirms this statement as it is the most specialized database in Computer Science in the dataset (second is dblp). It covers 67% Computer Science, 9% Engineering, 9% Mathematics, 4% Decision Science and 2% Social Sciences, 2% Business, Management and Accounting, and 2% Medicine.

The quantitative estimates of BOK were all plausible insofar as they reflected the textual descriptions of the six most specialized databases in the dataset. Accordingly, these validations show that the BOK method is capable of consistently quantifying subject coverage with reasonable margins of error. As a consequence, BOK can be rated as accurate in detecting coverage of highly and least prevalent subjects at specialized databases.

Any interpretation of these results must consider that using different subject classification systems that set disciplinary borders differently will rate the subjects’ exact relative and absolute coverage differently (see section Comparison of Scopus and WOS CC). For example, the relative greater importance of Medicine over Nursing in CINAHL Plus stems from BOK detecting a higher prevalence of keywords with an association with Medicine according to Scopus’ ASJC, rather than with an association with nursing. As the BOK method is employed consistently across all databases in the dataset, the ASJC interpretation is constant too. In the case of CINAHL Plus, this means that even if Nursing was valued at ‘just’ 16%, it still has by far the greatest relative coverage across all systems in the dataset. Accordingly, it does not mean that the BOK estimate is off by a margin, but that deviations also arise due to classification systems employing different logics in attributing categories. BOK offers an alternative interpretation of subject coverage, as it also detects coverage that is not natively supported by the classification regime of a database.

#### Specialized databases with very narrow subject focus

Estimates were drawn for the ERIC database via its standalone system that does not support verbatim queries and EBSCOhost that does. The estimates for both databases were highly correlated (0.997). For both systems, BOK method estimated ERIC’s coverage to 57/52% for Social Sciences, 13/17% for Psychology, 9/12% for Arts and Humanities, 8/7% for Medicine. Education Resources Information Center (2021) notes it *“being is a comprehensive, easy-to-use, searchable, Internet-based bibliographic and full-text database of education research and information.”* As there is no further textual information on its coverage, its journal list was reviewed. The 17 topics^5^ it covers are mostly attributable to the subject of Social Sciences and some, particularly ‘Counseling and Student Services’ to the field of Psychology, confirming the BOK estimation.

A similar analysis was performed for SPORTDiscus, which notes: *“it is the most comprehensive, bibliographic database covering sport, physical fitness, exercise, sports medicine, physical education, kinesiology, training, disabled persons, drugs, health, health education, biomechanics, movement science, injury prevention rehabilitation, physical therapy, rehabilitation, nutrition, exercise physiology, sport & exercise psychology, occupational health & therapy, public health and more.”* BOK estimated its relative coverage at 38% Health Professions, 26% Medicine, 13% Social Science, 6% Psychology, 3% Business, Management and Accounting, 3% Arts and Humanities, and 3% Nursing. It is interesting to see that even with both ERIC’s and SPORTDiscus’ narrow subject coverage, the BOK estimates seem plausible. This result is promising as it indicates the BOK method is robust, even for selected databases with a subject focus narrower than the ASJC classification.

## Discussion

This study presents a novel method to estimate the subject coverage of scholarly databases. The BOK method made it possible to rank 56 databases based on their relative and absolute coverage and to determine their level of specialization. These findings are particularly helpful as they both quantify a textual description of coverage but also facilitate comparisons of subject coverage. BOK estimates have been shown to detect subjects that are not described in coverage descriptions. For example, seekers of Psychology literature might be surprised to learn about the more than 13% Psychology coverage in ERIC, a database focused on education that does not expressly mention its Psychology coverage, albeit listing psychology-oriented journals in its content.

Comparability of the individual database estimates (i.e., internal validity) is rated as high due to the BOK method being consistently and rigorously applied across databases. This study aimed to determine subject coverage across databases with sufficient precision to inform database selection. For a researcher, it is largely irrelevant whether a system has 77% or 82% coverage in a specific subject; what is relevant is reliable coverage estimates compared to other databases. BOK does that particularly well.

Assessments of the external validity showed the levels of inaccuracies that should be accounted for when interpreting the estimates for multidisciplinary systems. For WOS, this deviation was calculated at an average of 19.6% across all 26 subject categories, a value relatively small compared to the little or imprecise information in existence on subject coverage of most databases. For specialized systems, external validity was assessed narratively by comparing BOK estimates with textual coverage statements; overall, all six highly-specialized databases and two narrowly specialized ones were estimated plausibly. Overall, while it is important to note that BOK provides estimates that will reflect actual coverage with a margin of deviation, the estimates can be considered robust and to offer plausible guidance for selecting databases.

### Search advice for each major academic search type

I discuss how researchers can utilize the estimated absolute and relative subject coverage of databases. Optimal database selection will depend on the goal of the researchers. Broadly speaking, academic researchers frequently have three different search goals: lookup, exploratory, or systematic (Gusenbauer & Haddaway, 2021).

#### Lookup searching

Lookup searches, where researchers know exactly what they are seeking, will require databases with high total absolute coverage (Table 2) or high absolute coverage in a specific discipline (Table 4), as the likelihood of a database covering the desired records is comparatively higher.

#### Exploratory searching

Exploratory searches benefit from high rates of absolute coverage of one or multiple potentially relevant subjects (Table 4), as serendipitous findings might occur in databases with a broader scope. However, if the goal is solely to explore a discipline or sub-discipline, then a database with high relative coverage (Table 5) might be the best choice. The fact that Google Scholar, as the largest database available, is used by most academics (Nicholas et al., 2017) engaged in lookup and exploratory searching indicates that users prefer comprehensiveness (Table 4) for these search types. In exploratory searching, the *search moves* are essential, consequently, search functionalities, such as citation searching or filtering, will play a greater role than they would in lookup searching.

#### Systematic searching

In systematic searches, where the goal is to identify all records on a subject, the optimal choice between high absolute and high relative coverage is not straightforward. As keyword queries can identify many irrelevant results when the subject focus is too wide, researchers need somehow to account for the problem. Researchers must either search specialized databases with high relative coverage (Table 5) or search multidisciplinary databases with high absolute coverage (Table 4) and limit the subject focus via subject-specific keywords or, when available, via subject filters or a controlled vocabulary. In all cases, users should search multiple databases when searching systematically (Konno & Pullin, 2020), including databases with specialized content (Table 5) (Bramer et al., 2017). Backward and forward citation searching of multidisciplinary databases with high rates of absolute coverage (Table 4) and a citation index helps to identify relevant records from a wide field of interest. Additional options are gray literature searching or hand-searching. The former can be particularly successful with larger databases that cover scholarly records of all kinds (Table 2).

The results of this study should also encourage researchers to use databases that are identified as relevant but not familiar. Using a variety of relevant databases will increase the number of identified relevant search results which is particularly beneficial in systematic searches (Konno & Pullin, 2020).

After we have considered the subject coverage of databases, it is important to remember that there are other questions researchers seeking optimal database selection should consider too. Here is a selection of those questions:

- Does the top-ranked database cover the *record type(s)* I seek? For example, most databases cover journal articles, but not all. For an overview of record type coverage, see Table 2.
- Is the *retrospective coverage* of the database adequate? For example, if you want to know about the origins of computer science, it is not advisable to choose arXiv, a database whose retrospective coverage starts in 1991. For information on retrospective coverage, see Table 2.
- Does my institution *subscribe to the database* that covers most records in my discipline? What is the share of *open access records* on the database? Paywalls considerably limit access to databases that provide specialized records in particular. However, just because a database is openly searchable does not mean its records are openly accessible. For an overview of paywalled versus open databases and their relative open access coverage, see Table 2.
- In the case of narrow search goals: does the top-ranked database also cover the most records for the *specific concept* I seek? For specific search goals, just a small number of records from an entire subject might be relevant. Some databases will cover this sub-topic more comprehensively than others. Researchers can assess the situation by conducting queries of their narrow concept(s) in several databases among those suggested by BOK estimates to contain the most records in the discipline (see Tables 4 or 5). To compare coverage results, the researcher must consistently apply queries with the same keywords and field codes across databases.
- Does searching a combination of databases yield better outcomes than searching a single one? Results show which databases are most comprehensive in single subjects. Depending on search goals (lookup/exploratory/systematic searching), it will make more sense to search a single database, multiple multidisciplinary ones, or multiple specialized ones. Aggregator systems (e.g., Web of Science, ProQuest, EBSCOhost) in particular permit searching multiple specialized databases at once to balance recall and precision.
- Does the search system support the *search heuristics* I want to perform? For example, not all search systems allow database access via Boolean queries, citation searching, filtering, or controlled vocabularies. It is important that users assess search functionalities relevant to their search goals of databases with good coverage also provide search functionalities relevant to their search goals. An in-depth analysis of approximately half of the systems analyzed in this study can be found in Gusenbauer & Haddaway, 2020.

### Comparison of the coverage of Scopus and WOS CC

To illustrate how BOK estimates should be interpreted in light of existing assessments of subject coverage, I discuss the findings for both Scopus and WOS CC. A recent literature review comparing both systems has called them *“The Titans of Bibliographic Information in Today’s Academic World”* (Pranckutė, 2021). As institutions must pay substantial fees to access these paywalled systems, particular attention has recently been directed at their disciplinary coverage, among other important characteristics (e.g. Aksnes & Sivertsen, 2019; Bakkalbasi et al., 2006; Chadegani et al., 2013; Harzing, 2019; Harzing & Alakangas, 2016; Kousha & Thelwall, 2008; Martín-Martín et al., 2018a, 2018b, 2021; Meho & Yang, 2007; Mongeon & Paul-Hus, 2016; Moskaleva & Akoev, 2019; Singh et al., 2021; Vera-Baceta et al., 2019; Vieira & Gomes, 2009; Visser et al., 2021).

Harzing and Alakangas (2016, p. 788) noted that *“Web of Science and Scopus provide fairly similar results,”* based on a review of the literature up to 2015. Recently, Pranckutė (2021, p. 7) summarized previous findings to show *“better Scopus coverage of all major disciplines when compared to WoS.”* BOK estimates, assessing coverage in 2021, plausibly update both these statements and offer a more nuanced view of their coverage. Both WOS CC and Scopus probably have unique merits because (1) their ADS are almost identical,^6^ yet (2) their coverage only overlaps to a certain extent. Previous studies found that both databases have significant proportions of unique records (Martín-Martín et al., 2018b, 2021; Visser et al., 2021), a finding substantiated by the BOK results. While BOK does not look at individual records, it is capable of detecting overlap at an aggregate level. The BOK estimates are derived from using the same keywords and query settings across databases. Accordingly, if indeed both databases overlapped to the greatest degree, the BOK estimates would show this by identifying similar keyword-based query results (QHCs) for both databases. Internal validity assessments show that BOK estimates work well in identifying whether systems access the same records. For example, Medline accessed by Ovid, WOS, and EBSCOhost were found to have very similar coverage (see Fig. 2). Accordingly, differences in BOK estimates between Scopus and WOS CC will be due to a significant share of *unique* records available in each database and the relative differences in disciplinary coverage of those records. Unlike sampling-based studies, BOK is, however, unable to determine the extent of the (non-)overlap.

#### How does BOK estimate Scopus versus WOS CC coverage?

**Fig. 3:**
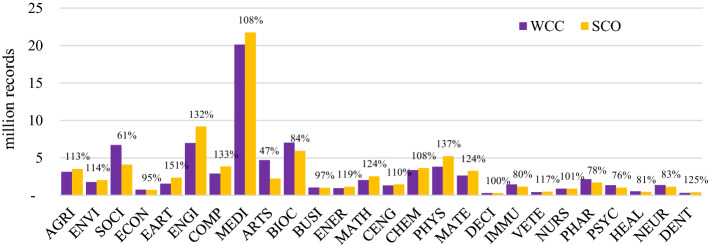
Comparison of absolute subject coverage between Scopus and WOS CC (based on single-attribution; %-values show coverage of Scopus versus WOC CC)

In this study, the results of Scopus’ coverage are precise, as they are not derived from estimates, but rather from direct queries based on the ASJC subject classification of Scopus. The WOS CC data, and the data from all other databases in this study, is based on BOK estimates relying on word frequencies provided by Scopus. Figure 3 compares the absolute subject coverage data for Scopus and WOS CC, a selection of the data illustrated is in Table 4. The comparison shows that Scopus only covers 47% of the records in Arts and Humanities and only 61% of those in Social Sciences that WOS CC does. Conversely, BOK finds Scopus’ coverage is notably superior in Physics and Astronomy (137% of WOS CC), in Earth and Planetary Sciences (132% of WOS CC), in Computer Science (133% of WOS CC), and in Engineering (132% of WOS CC).

**Fig. 4:**
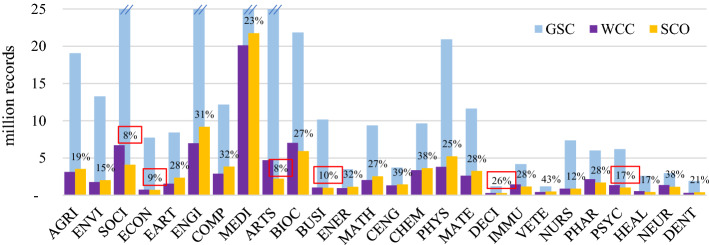
Comparison of absolute subject coverage between Scopus and WOS CC (based on single-attribution; %-values show the coverage of Scopus versus Google Scholar; Social Sciences and Humanities subjects highlighted in red)

Assuming Google Scholar’s index is the most comprehensive collection of academic literature (Gusenbauer, 2019), WOS CC and Scopus only cover a small portion of that. BOK estimates confirm that both WOS CC and Scopus tend to cover more from the Life Sciences, Physical Sciences, and Health Sciences and less from the Social Sciences and Humanities than Google Scholar does (Pranckutė, 2021; Singh et al., 2021). Figure 4 shows Scopus’ coverage compared to Google Scholar, with Social Science and Humanities subjects highlighted in red. It shows how Scopus covers relatively more from the Engineering, Computer Science, Energy, Chemical Engineering, Chemistry, Veterinary, and Neuroscience fields, whereas Social Sciences, Economics, Arts and Humanities, and Business and Management are covered to only a smaller degree. A notable exception is Decision Science, a subject that is covered almost as well as Computer Science or Mathematics—subjects it is more closely related to than the subjects in Social Science and Humanities.

### How to compare BOK results against other studies’ results

Some BOK findings confirm previous findings and some contradict them. Previous examinations are not homogeneous in their assessments of the coverage of Scopus and WOS CC (Pranckutė, 2021). The extent to which BOK estimates reflect actual subject coverage in databases (i.e., external validity) will also depend on what one considers ‘actual coverage’. Judging external validity also depends on the reference point, that is, the methodical decisions shaping a study. Those factors include the choice of subject classification system, sampling-based versus full analysis, retrospective coverage, journal- versus document-level analysis. It is also important to consider the variations in those decisions across studies. Four factors that will contribute to different subject coverage results across studies (not within studies) are discussed below in greater detail: (1) differences in institutional subscriptions of WOC CC, (2) differences in analysis procedures of subject classification, (3) differences in subject classification systems, and (4) differences in subject attribution.

#### Differences in institutional subscriptions of WOC CC

The first factor that needs to be accounted for when comparing WOS CC to other databases is the issue of differences in institutional coverage. That issue necessitates always considering WOS CC coverage results in light of the unique access situation of the investigating researcher. For example, the results of Visser et al. (2021) are difficult to compare with as their subscription starts in 1980; the subscription in this study starts considerably earlier for most indices. The version of the WOS CC included in this study is comprehensive in that it covers almost all of the records WOS CC provides in its full version. Only some minor indexes are not included in this study’s analysis of WOS CC (see Table 2). To enhance the assessment of WOS CC, this study includes all major sub-indexes of the WOS CC also individually, each index in its full retrospective coverage.

#### Differences in analysis procedures of subject classification

Second, differences occur in how a single document is determined to be attributable to a subject. BOK estimates are accurate insofar as they count each document that matches a highly-specialized keyword that represents a subject. These keywords are representative of the larger proportion of coverage of an entire subject. Inferences can be made about the entire database because the likelihood of a highly-specialized keyword being attributable to a document is known. While those inferences will not be exact, BOK estimates have the merits of attributing subject-coverage to each individual document in the database.

Sampling-based approaches make assumptions based on a sample of documents from the total. A document’s subject is then determined, either directly based on the subject(s) the journal is attributed to (e.g., Harzing & Alakangas, 2016) or indirectly where the subject attribution of a citing document is determined via the subject attribution of the seed document (e.g., Martín-Martín et al., 2021). Differences in how samples of documents are drawn, and on how directly subjects are attributed to documents will determine the comparability and the precision of results.

#### Differences in subject classification systems

Third, results from this study can only be directly compared to studies that also adopt the ASJC classification system at the level of analysis of this study. To the best of my knowledge, no studies use ASJC and compare Scopus and the WOS CC. Every classification system will demarcate subjects differently, even when the subjects are titled the same way. Previous assessments of the subject coverage of Scopus and WOS CC used *Scopus’ ASJC at the five-category level* (Visser et al., 2021), *Google Scholar categories at the 252 and eight-category levels* (Martín-Martín et al., 2018a, 2018b, 2021), the *National Science Foundation classification at the four-category level* (Mongeon & Paul-Hus, 2016), *WOS classification at the five-category level* (Singh et al., 2021) or *not closer specified classifications at the four-category level (*Aksnes & Sivertsen, 2019*) and self-specified classifications at the five-category level* (Harzing & Alakangas, 2016). As it is difficult to identify the best classification system, a multitude of different approaches might encourage better research classifications by comparing and learning (Wang & Waltman, 2016). Nevertheless, a drawback of scientometric studies using different classification systems is that comparing them will always be a vague process. For the method of BOK, it did not make sense to adopt one of the previously used classification systems (see section ‘Selection of reference database and its subject classification: Why ASJC by Scopus?’).

#### Differences in subject attribution

Fourth, an important question is how subject coverage is determined in terms of multi- or single-attribution. Most of the studies found comparing the subject coverage of Scopus and WOS CC assume one record is unequivocally attributable to a single subject: For example, Mongeon and Paul-Hus (2016) assume journals are categorizable into one of four disciplines, and Martín-Martín et al. (2021) assume cited documents share the same single subject the seed document was classified against under the Google Scholar categories. Nevertheless, records are often attributable to more than one subject. According to the WOS CC classification system, one record is on average attributable to 1.33 subjects, in arXiv the figure is 1.12, and in Scopus it is 1.59.

The absolute and relative subject coverage rates determined via BOK are based on fractional counting (Perianes-Rodriguez et al., 2016; Visser et al., 2021), which I refer to as single-attribution. Consequently, if a record is attributed to both Mathematics and Decision Sciences each subject is awarded half of one point, so the sum of subjects equals the sum of records. This study relied on single-attribution, as multi-attribution calculations of estimated databases would mean overly inflating their coverage. For example, for highly-specialized databases with excellent coverage in a single subject, calculating multi-attribution might mean that one subject has 100% coverage. Nevertheless, 100% coverage of a single subject is very unlikely, as there are likely to always be records from other subjects available in databases. The choice of single-attribution values does not limit comparability across databases as the assumptions are applied equally across all databases. Overall, the logic of assigning one or multiple subjects to a single document will, however, impact the results of subject coverage assessments. The reader needs to be aware that due to single-attribution, absolute subject coverage values estimated in this study should be interpreted as indicating that a database covers at least that number of records. The actual number of records in a specific subject will likely be higher, given that most records are attributable to multiple subjects.

### Contribution of the BOK method

#### BOK as an additional method for coverage estimation

The BOK method has several advantages over contemporary scientometrics methods in assessing database coverage. BOK is compatible across many databases (high internal validity) and has a low marginal cost of updating and adding new estimates. Specifically, BOK can help continuously analyze databases that are relatively new and regularly updated, such as Lens or scite.

Its merits make BOK an ideal complement to existing sampling-based methodologies to assess database coverage. Primarily, BOK estimates are an efficient way to estimate absolute subject coverage values of entire databases, information that is typically missing in sampling-based studies because they often calculate overlap- or the coverage values of a specific sub-sample. Here BOK’s external validity is considerably improved by calibrating (normalizing) absolute coverage values based on the absolute database size—data that in most cases can be considered accurate. As the ADS vary greatly across databases—from close to 1 million to almost 400 million records—the absolute subject coverage estimates of BOK will reflect those differences. Accordingly, this kind of normalization ensures that estimates are within a certain absolute margin of deviation anchored at the ADS.

While BOK can provide a picture of the coverage of many databases, sampling-based studies can give a detailed view of specific databases (e.g., Martín-Martín et al., 2018a, 2018b, 2021; Visser et al., 2021; Walters, 2007). For example, the BOK method can provide information on databases with similar high subject coverage or information on databases that seem to overlap considerably (e.g., see Fig. 2: EPM, PMD, MED1-3, EMB, MET). Sampling-based methods could then analyze the overlap of databases with regard to specific types of records. In this example, analyzing the coverage of Europe PMC, PubMed, Medline (via Ovid, WOS, EBSCOhost), Embase, and the newly released/discontinued Meta would give more insights into their areas of overlap and levels of uniqueness.

The successful application of BOK in this study should also promote its application to different settings. BOK could be extended to analyze subject coverage of languages, authors, specific topics, and other criteria. It is also possible to investigate coverage at a sub-disciplinary level to determine an even more granular picture of what specific subtopics are covered or otherwise in specific databases. Furthermore, librarians might use the method to investigate differences between subscription packages offered by WOS, ProQuest EBSCOhost, or Ovid. Such an investigation might, for example, reveal the different coverage options of the WOS Core Collection. Given its broad applicability, it could be used to compare many smaller niche databases that often remain in the shadow of larger databases that promise superior coverage. Making these systems readily comparable can provide a promising way forward to shed light on databases and systems that have been too long overlooked.

#### The use of QHC as a measure of bias in search queries in general

BOK uses QHC as its underlying data collection method. If QHCs are inaccurate due to inexact keyword matching, these issues will occur for any search of the database in question. All users who access the database will find their keyword queries interpreted in some non-transparent way so that the search results obtained are biased. Semantic search systems such as Google Scholar, Microsoft Academic, or Semantic Scholar are proponents of such search functionalities. Microsoft Academic noted in its FAQs: *“Traditional search engines rely mostly on keyword matching. Usually, they match the keywords you type in the search field with words found in the indexed content. The accuracy of the search results depends on the quality of the keywords you type, which puts the responsibility of a successful search on the user (Microsoft Academic,* 2021*).”* As more systems take responsibility for articulating search goals away from users, the systems will also introduce bias and opaque algorithms that impede transparent, reproducible data collection. QHCs reflect what is actually available to the user via queries. Other data that is not retrievable via queries—the main way of identifying records at most search systems—will probably not emerge and thus will not be accessible. For example, in several cases, official information regarding retrospective coverage is inaccurate. Manually verifying retrospective coverage for these systems via QHCs showed that many have greater or lower retrospective coverage than is reported (see Table 2).

QHC method can illustrate the limitations of limited search functionality (Gusenbauer & Haddaway, 2020) and database descriptions. Further, it can illustrate how query results can be even more accurate than official information, and in its application of BOK the method can be used to get a more accurate picture of the coverage of a database. QHCs were used in this study as an efficient tool to determine not only subject coverage but also ADS, retrospective coverage, English, and open access coverage (see Table 2).

### Limitations

While the BOK method provides good and robust estimates of subject coverage, the results should be interpreted with an eye on some limitations.

#### Language coverage

As the selected keywords are only in English, they will only identify English records. If the underlying dataset’s language composition is substantially different from that of Scopus, the estimates will be somewhat biased. Biases will occur when a keyword is used in multiple languages or shares the name with a prominent author. Keyword-specific differences were alleviated by selecting suitable keywords yet cannot be fully ruled out. To alleviate language bias, this study focused on databases with a majority of English content. Some variation in language composition is acceptable when we assume that the relative subject composition of the underlying dataset is similar between English and non-English records.

Given that English acts as the lingua franca of science communication, the BOK method applies to most popular bibliographic databases researchers use today. Nevertheless, there might be benefits to searching for and including non-English records in research, depending on the purpose of the research. For example, in the realm of evidence synthesis, the results of quantitative syntheses are changed by using additional non-English sources (Walpole, 2019). Other research found, however, that conclusions from evidence synthesis in health sciences remained similar for a sub-sample of all-English sources (Nussbaumer-Streit et al., 2020). Even though the effect of including non-English studies in evidence synthesis is not entirely clear, what is always true, however, is that including non-English databases will increase the variety of evidence in literature searches (Konno & Pullin, 2020) and thus improve outcomes. While databases in this study already partially cover non-English records, researchers seeking non-English records too will probably need to search other non-English databases.

#### Quality of underlying records

The estimations of databases’ subject coverage provided in this study do not indicate the *quality* of the underlying records. It is important to note that some databases focus on providing peer-reviewed, published records (e.g., Scopus or Web of Science), while others (also) include data of all types and quality (e.g., Core or BASE). Both these database types have their merits. The overall quality of records is higher in the former, while the latter might be more comprehensive and also include the gray literature important for quantitative analyses (Haddaway et al., 2015). The current research only compares databases that exclusively or at least mostly cover the various forms of scholarly records (see Table 2). That choice was made to ensure the consistency of the BOK method.

Another area of bias is the number of duplicate records and other database errors a database has. For example, duplicate rates vary from almost zero (0.00–0.05%) for Web of Science on various databases to almost five percent (1.0–4.8%) for Google Scholar (Haddaway et al., 2015; Orduna-Malea et al., 2017). Another study found Google Scholar’s duplication rate to be 4% and Scopus’ rate to be 2% (Moed et al., 2016). Data issues and particularly duplicate records are likely to be present in all databases to some degree. For example, a study found that WorldCat and other (non-)academic databases contain a number of duplicate records (Wilder & Walters, 2021). While the exact duplication rates are difficult to assess, duplication rates are likely to be lower for curated databases than for crawler-based ones. Google Scholar’s pre-eminent position in terms of superior subject coverage across most subjects is not at risk, even when factoring in a 5% duplicate rate. This secure position is even more likely as the runners-up (BASE, Microsoft Academic, Semantic Scholar, Core, Lens) are also likely to have similar duplication issues. Overall, these differences should be taken into account when selecting databases based on coverage preferences: the higher the duplicate rate, the more BOK estimates will overvalue *absolute* subject coverage. The *relative* shares of disciplinary coverage are unlikely to be particularly affected by duplicates.

#### Quality of ASJC classification

The liberal approach evident in Scopus with regard to assigning subjects to its records has been criticized (Mongeon & Paul-Hus, 2016; Wang & Waltman, 2016). This study not only confirms these previous findings but also shows subjects are attributed unevenly. The subjects with the least overlap were found to be Medicine (70% unique), Dentistry (64% unique), and Veterinary (56% unique), while the greatest overlap occurred in Decision Sciences (5% unique), Materials Science (10% unique) and Chemical Engineering (10% unique). The overlap percentages raise the question of whether categories are sufficiently unique particularly in the case of Decision Sciences and other highly overlapping categories. Conversely, larger categories might benefit from being divided. The issue of unevenly unique subject categories was also noticeable at the keyword level, where it was most difficult to find precise keywords for categories with the most overlap. That issue could negatively affect the accuracy of the estimates for those subjects. The differences in subject overlaps are addressed by using precision thresholds in calculating BOK estimates. Overall, this limitation illustrates the need to scrutinize the qualities of the subject classification systems in general and ASJC in particular.

## Conclusion

This study acknowledges the substantial need for information seekers to know the subject coverage of databases to be able to compare them when designing an optimal search strategy. That great need is particularly illustrated by the number of scientometric studies, and the significant attention they receive in terms of citations and other metrics. The results of this study should encourage information seekers to compare subject coverages across 56 academic databases based on a consistent and robust method. Database choices should be re-evaluated, particularly in terms of which go-to systems provide superior coverage and in how far the ‘new players’ can offer viable search alternatives. I believe data based on the BOK method significantly improves the database choice of information seekers across disciplines, particularly as even more databases might be compared in the future. Improved database choice options will encourage researchers to search for the most suitable systems and access more relevant records.

Validation showed BOK estimates are accurate where the estimates are most helpful: for multidisciplinary databases. Specialized databases are somewhat known to represent specific subjects, but the focus of multidisciplinary databases is more opaque. Accordingly, a medical scientist will most likely be aware of the high coverage of Medicine topics in PubMed, but probably be unaware of the coverage of Medicine in Google Scholar or the new(ly discontinued) Meta database. The former is estimated to cover by far the most Medicine records and the latter also has significantly greater absolute coverage than PubMed or Medline, while having similar relative coverage, which might surprise many dedicated PubMed users.

Given the vague information that currently guides database choice, the BOK method offers a significant step toward effective literature searching. BOK can be relatively easily extended to more databases and continuously updated to track developments over time. While this study aimed to inform database choice, the ideal database is not characterized by coverage alone. A recent study points to the importance of the differences in search functionality between databases and how they are important for specific search requirements (Gusenbauer & Haddaway, 2020). Overall, the optimal choice of database system (*what)*, is determined by *why* researchers are searching (the goals) and *how* they want to search (the heuristics)—the so-called ‘search triangle’ (Gusenbauer & Haddaway, 2021).

### Future directions

Further improvements in the estimation of subject coverage of databases might come either via an improvement of BOK or an update of the sampling method. BOK could be further improved when databases allow more accurate and comprehensive searches via their search interfaces. Specifically, Boolean searches with multiple keywords and keyword combinations would improve the accuracy of BOK. At the moment, however, such functionality is only available for a fraction of the systems covered in this study. Second, sampling-based approaches could be improved if the citation data of databases became more readily available. Currently, database providers guard that information, making comparisons across databases extremely labor intensive. If researchers had access to all records and their full text, record-level subject classification would be the best way forward. Unfortunately, that endeavor has received a serious blow with the discontinuation of Microsoft Academic. In the meantime, BOK method is a good compromise to help improve database choice.

## Figures and Tables

**Table 9:**
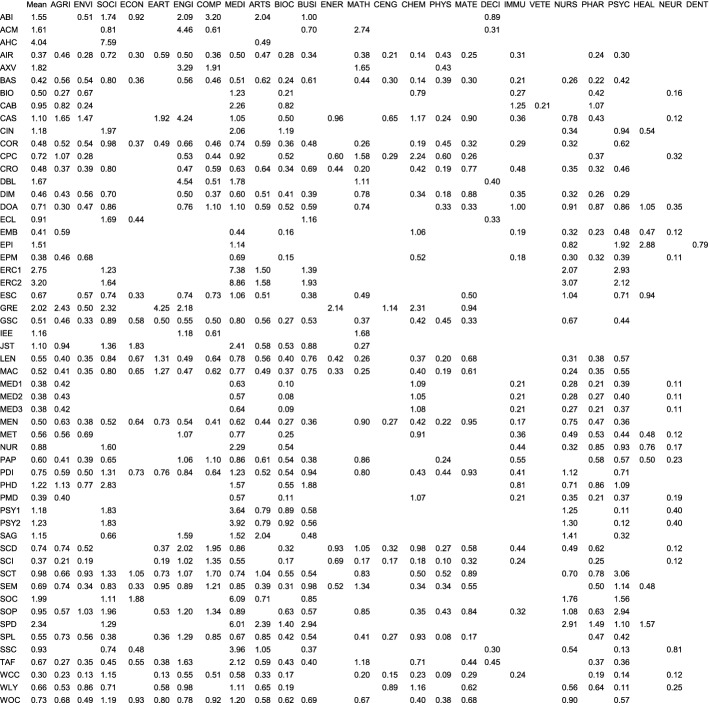
RIQR2.5 for 55 databases in the sample (Scopus not included as its values are not based on BOK estimates; abbreviations see last page of Table 2)

**Table 10:**
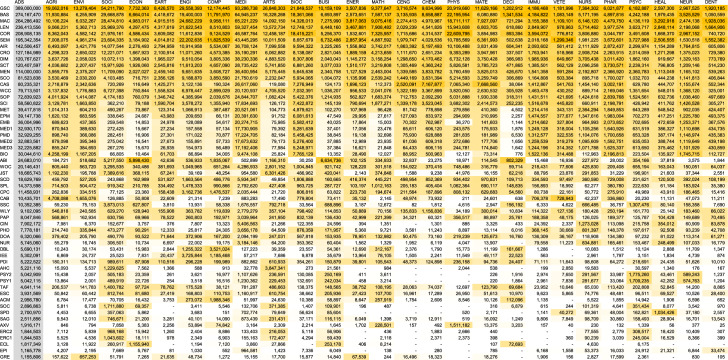
Absolute coverages of the 56 databases for 26 subjects, sorted by absolute database size (based on single-attribution; darker coloring reflects more absolute coverage compared to other databases; abbreviations see last page of Table 2)

**Table 11:**
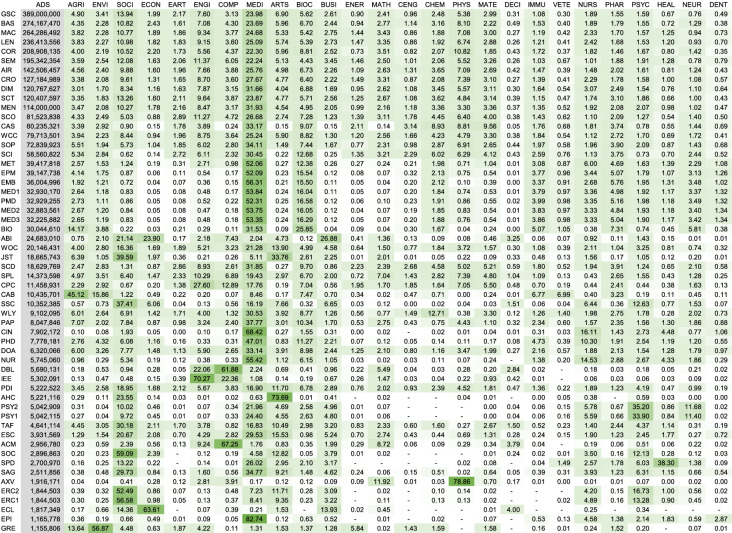
Relative coverages of the 56 databases for 26 subjects, sorted by absolute database size (based on single-attribution; darker coloring reflects more relative coverage; abbreviations see last page of Table 2)

## Appendix

See Tables

**Table 7:** Important concepts in this study

Concept	Definition	Example
Search system	The system that assesses a database. Can be a search engine, an aggregator or some other type	Google Scholar (the search engine), PubMed (bibliographic database), Web of Science (Aggregator)
Database	The underlying bibliographic database accessed via a search system	Google Scholar (the index), WOS CC
Reference database	Scopus was used as the reference database using its ASJC System and the information on the subject attribution	
Control database	WOS CC was used as the control database to verify the accuracy of the estimates. Exact indices used see footnote 1	
Query hit count (QHC)	Number of hits specific keyword query	1,239,041 hits for “blight”
Query subject count (QSC)	Number of hits from a specific subject of a specific query	11,123 for Mathematics 15,456 for Physics and Astronomy
BOK	Novel analytic method based on specific keyword queries allowing the assessment of subject coverage of a large number of bibliographic databases in academia	
Precision	The proportion of records identified on a specific subject of the total of records identified across all subjects (QSC/sum of QSCs)	With 90.3% precision, “branes” is the most precise keyword for Physics and Astronomy using the title field code
Recall	The proportion of subject-attributed records of all records available for the subject, identified by a single keyword query	The recall of “branes” is 0.06% for Physics and Astronomy using the title field code
Absolute database size (ADS)	The absolute number of records on a database determined via official information or QHC. Every record is counted only once	
Multi-attribution (of a database)	Number of subject categories that are attributed to a single record	On average, Scopus records are attributed with 1.59 subject categories each. 57% have only a single category
Relative Interquartile Range (RIQR)	(Third quartile—first quartile)/median	RIQR were between 0.31 and 14.70 in this dataset, whereas a low value indicates homogeneous estimates, and a high value indicates heterogeneity

7,

**Table 8:** The BOK used to determine QHCs (for each of the 26 subject categories 14 keywords, totaling 364 keywords)

**Agricultural and Biological Sciences**	Blight	Coleoptera	Genera	Germination	Habitat	Lepidoptera	Mycorrhizal	Neotropical	Phylogeny	Pollination	Postharvest	Predation	Soil	Tillage
**Arts and Humanities**	Aristotle	cinema	comedy	externalism	god	historiography	literary	medieval	metaphysics	naturalism	philosophy	poetry	shakespeare	theology
**Biochemistry, Genetics and Molecular Biology**	Actin	arabidopsis	chromatin	drosophila	histone	kinase	mitochondria	nucleosome	phosphatidylinositol	phosphorylation	reticulum	ribosomal	transcriptional	ubiquitin
**Business, Management and Accounting**	Ambidexterity	brand	business	corporate	customer	effectuation	employee	entrepreneurial	firms	hospitality	ifrs	interfirm	loyalty	marketing
**Chemical Engineering**	Ammoxidation	epoxidation	fluidization	hydrodeoxygenation	hydrodesulfurization	hydroisomerization	hydrotreating	hzsm	methanation	nimo	spouted	syngas	tropsch	zeolite
**Chemistry**	Alkynes	aryl	bridged	carbene	catalyzed	cycloaddition	dinuclear	enantioselective	heterocycles	intramolecular	ketones	nucleophilic	palladium	stereoselective
**Computer Science**	Android	cache	compiler	hashing	mapreduce	middleware	multicast	multithreaded	prefetching	recommender	software	virtualization	watermarking	wireless
**Decision Sciences**	Backlogging	balking	bicriteria	capacitated	flowshop	lotsizing	multiserver	newsvendor	queueing	queues	retrial	rostering	tardiness	uncapacitated
**Dentistry**	caries	dental	denture	edentulous	endodontic	gingival	intrabony	mandibular	molars	orthodontic	overdentures	periodontal	protaper	restorations
**Earth and Planetary Sciences**	Craton	cretaceous	crustal	geochemistry	geochronology	mafic	magmatism	metamorphism	neoproterozoic	orogenic	petrogenesis	subduction	tectonic	zircon
**Economics, Econometrics and Finance**	Countercyclical	dsge	fiscal	hedge	interbank	liquidity	macroeconomics	monetary	oligopoly	premia	spillovers	unemployment	volatility	wage
**Energy**	Acidizing	asphaltene	downhole	drilling	exergoeconomic	exergy	gasification	geothermal	hvdc	microgrid	proppant	renewables	shale	wellbore
**Engineering**	Antennas	brushless	capacitor	cdma	cmos	converters	inverters	machining	microstrip	mimo	sensorless	stator	transformer	wideband
**Environmental Science**	Bioaccumulation	catchment	groundwater	hydrological	leachate	pollution	riparian	runoff	sediment	sludge	stormwater	streamflow	wastewater	wetlands
**Health Professions**	Audiology	bestest	chiropractic	hamstring	interexaminer	isokinetic	judo	kinesio	lumbopelvic	optometry	physiotherapist	slagle	taping	volleyball
**Immunology and Microbiology**	Capsid	emended	ictv	leishmania	listeria	monocytogenes	serovar	simian	subgenomic	trypanosoma	vaccinia	virion	virology	virulence
**Materials Science**	Alloys	austenitic	carbide	ceramics	coatings	composites	graphene	mullite	nitride	polymers	pressureless	sintering	stainless	superalloy
**Mathematics**	Adic	algebras	banach	cauchy	cohomology	elliptic	manifolds	mappings	nonexpansive	polynomials	quasilinear	riesz	semilinear	sobolev
**Medicine**	Aortic	artery	carcinoma	coronary	disease	hypertension	myocardial	percutaneous	postoperative	pulmonary	renal	resection	surgery	transplantation
**Neuroscience**	Accumbens	amygdala	axotomy	cortex	gabaergic	gyrus	hippocampal	interneurons	nmda	prefrontal	striatal	synaptic	tegmental	thalamus
**Nursing**	Aides	baccalaureate	braden	breastfeeding	caregivers	caring	dietetics	hospice	midwifery	nicu	nurse	nursing	nutrition	palliative
**Pharmacology, Toxicology and Pharmaceutics**	Adrenoceptor	anxiolytic	bioactivation	excipients	glucuronidation	hepatotoxicity	medicinal	microsomes	mucoadhesive	pharmacokinetic	pharmacology	tetrachlorodibenzo	toxicology	transdermal
**Physics and Astronomy**	Baryon	boson	branes	decays	gluon	hadron	hadronic	higgs	meson	neutrino	nucleon	pion	quark	supersymmetric
**Psychology**	Esteem	eyewitness	intergroup	mindfulness	narcissism	neuropsychology	neuroticism	parenting	preschoolers	psychologists	psychopathy	psychotherapy	rumination	stereotype
**Social Sciences**	Democracies	electoral	federalism	geography	journalism	nationalism	parties	peace	politics	rights	sociology	sovereignty	teacher	transnational
**Veterinary**	Alfaxalone	brachycephalic	foals	greyhounds	horses	hyperadrenocorticism	lameness	laminitis	mares	ovariohysterectomy	racehorses	spaniels	thoroughbred	veterinarians

8, 9, 10 and 11.

## Footnotes

1 For 22 of the 56 databases language metadata is not available. It is assumed that these databases—most of them are non-proprietary—include at least 50% English content. All these systems’ (default) search interface language is English which is regarded as a sign for English-focus. The largest (non-proprietary) database is Google Scholar. Given its English content share is estimated at 65%, it can be assumed—at least for other large, non-proprietary systems—that their share of English content is also significantly exceeding 50%.

2 *NOT INCLUDED DATABASES:* AMiner (no QHC data above 10,000 hits); CiteSeerX (QHC data not plausible); ClinicalTrials.gov (Clinical trials); Cochrane Central Register of Controlled Trials (Clinical trials); LI-LACS (via Virtual Health Library; Clinical trials); Transport Research International Documentation (No QHC data above 15,000 hits); WorldWideScience (QHC data not plausible); WorldCat was only included in part (thesis/dissertation).

3 Science Citation Index Expanded (SCI-EXPANDED). Social Sciences Citation Index (SSCI). Arts & Humanities Citation Index (A&HCI). Conference Proceedings Citation Index- Science (CPCI-S). Emerging Sources Citation Index (ESCI). BIOSIS Citation Index.

4 To simplify, the ‘Medline’ name was adopted from Ovid, Web of Science, and EBSCOhost describing their coverage of what is most likely all of PubMed (they have the same ADS). It is important to note that PubMed includes Medline, yet also other databases. A detailed description of PubMed's coverage can be found here: https://www.nlm.nih.gov/bsd/difference.html

5 Adult, Career, and Vocational Education, Assessment and Evaluation, Community Colleges, Counseling and Student Services, Disabilities and Gifted Education, Educational Management, Elementary and Early Childhood Education, Higher Education, Information and Technology, Languages and Linguistics, Reading, English, and Communication, Rural Education and Small Schools, Science, Mathematics, and Environmental Education, Social Studies and Social Science Education, Teaching and Teacher Education, Urban Education, General.

6 WOS CC coverage varies from institution to institution. However, the coverage of about 80 million records reported in this study likely falls only slightly short of its maximum full coverage. As of December 2021, coverage assessments with the methods described in Gusenbauer (2019) show that the full version of WOS CC covers only slightly fewer records than Scopus.

## References

[CR1] Aksnes DW, Sivertsen G (2019). A criteria-based assessment of the coverage of Scopus and Web of Science. Journal of Data and Information Science.

[CR2] Allen Institute for Artificial Intelligence. (2022). *Why Semantic Scholar?: Multidisciplinary scope*. Retrieved January 17, 2022, from https://www.semanticscholar.org/about/librarians.

[CR3] American Chemical Society. (2022). *CONTENT OF SCIFINDER*^*n*^. Retrieved January 17, 2022, from https://www.cas.org/solutions/cas-scifinder-discovery-platform/cas-scifinder/content.

[CR4] arXiv. (2021). *arXiv.org*. Retrieved July 18, 2021, from https://arxiv.org/.

[CR5] Association for Computing Machinery. (2022). *The ACM guide to computing literature*. Retrieved January 17, 2022, from https://libraries.acm.org/digital-library/acm-guide-to-computing-literature.

[CR6] Bakkalbasi N, Bauer K, Glover J, Wang L (2006). Three options for citation tracking: Google Scholar, Scopus and Web of Science. Biomedical Digital Libraries.

[CR7] Bielefeld Academic Search Engine. (2021). *What is BASE?* Retrieved July 15, 2021, from https://www.base-search.net/about/en/.

[CR8] Bornmann L (2018). Field classification of publications in Dimensions: A first case study testing its reliability and validity. Scientometrics.

[CR9] Bramer WM, Rethlefsen ML, Kleijnen J, Franco OH (2017). Optimal database combinations for literature searches in systematic reviews: A prospective exploratory study. Systematic Reviews.

[CR10] Bryan M, Cecchetti S (1993). The consumer price index as a measure of inflation.

[CR11] Chadegani AA, Salehi H, Yunus M, Farhadi H, Fooladi M, Farhadi M (2013). A comparison between two main academic literature collections: Web of Science and Scopus databases. Asian Social Science.

[CR12] Clarivate Analytics. (2021). *Web of Science: Arts & Humanities Citation Index*. Retrieved August 16, 2021, from https://clarivate.com/webofsciencegroup/solutions/webofscience-arts-and-humanities-citation-index/.

[CR13] Clarivate Analytics. (2022a). *BIOSIS citation index*. Retrieved January 17, 2022a, from https://webofscience.help.clarivate.com/en-us/Content/biosis/biosis-citation-index.htm.

[CR14] Clarivate Analytics. (2022b). *Data citation index help*. Retrieved January 18, 2022b, from https://images.webofknowledge.com/WOKRS526R4/help/DRCI/hp_subject_category_terms_tasca.html.

[CR15] Clarivate Analytics. (2022c). *MEDLINE on Web of Science*. Retrieved January 17, 2022c, from https://clarivate.com/webofsciencegroup/solutions/webofscience-medline/.

[CR16] Clarivate Analytics. (2022d). *Web of Science Core Collection*. Retrieved January 17, 2022d, from https://clarivate.com/webofsciencegroup/solutions/web-of-science-core-collection/.

[CR17] Clarivate Analytics. (2022e). *Web of Science: Conference proceedings citation index*. Retrieved January 17, 2022e, from https://clarivate.com/webofsciencegroup/solutions/webofscience-cpci/.

[CR18] Clarivate Analytics. (2022f). *Web of Science: Emerging sources citation index*. Retrieved January 17, 2022f, from https://clarivate.com/webofsciencegroup/solutions/webofscience-esci/.

[CR19] Clarivate Analytics. (2022g). *Web of Science: Science citation index expanded*. Retrieved January 17, 2022g, from https://clarivate.com/webofsciencegroup/solutions/webofscience-scie/.

[CR20] Clarivate Analytics. (2022h). *Web of Science: Social sciences citation index*. Retrieved January 17, 2022h, from https://clarivate.com/webofsciencegroup/solutions/webofscience-ssci/.

[CR21] CORE. (2021). *Data, data, data*. Retrieved July 25, 2021, from https://core.ac.uk/data.

[CR22] Crossref. (2022). *Crossref*. Retrieved January 17, 2022, from https://search.crossref.org/.

[CR23] Da Teixeira Silva JA, Tsigaris P, Erfanmanesh M (2020). Publishing volumes in major databases related to Covid-19. Scientometrics.

[CR24] dblp computer science bibliography. (2022). *What is dblp?* Retrieved January 17, 2022, from https://dblp.org/faq/What+is+dblp.html.

[CR78] de Moya-Anegón F, Chinchilla-Rodríguez Z, Vargas-Quesada B, Corera-Álvarez E, Muñoz-Fernández FJ, González-Molina A (2007). Coverage analysis of Scopus: A journal metric approach. Scientometrics.

[CR25] Dimensions. (2022). *Dimensions: Breadth of data*. Retrieved January 17, 2022, from https://www.dimensions.ai/products/free/.

[CR26] DOAJ. (2022). *About DOAJ*. Retrieved January 17, 2022, from https://doaj.org/about/.

[CR27] EBSCOhost. (2021a). *APA PsycInfo*. Retrieved August 16, 2021a, from Paywalled URL.

[CR28] EBSCOhost. (2021b). *CINAHL Plus*. Retrieved August 16, 2021a, from Paywalled URL

[CR29] EBSCOhost. (2021c). *EconLit*. Retrieved August 16, 2021b, from Paywalled URL.

[CR30] EBSCOhost. (2021d). *ERIC*. Retrieved August 20, 2021d, from Paywalled URL.

[CR31] EBSCOhost. (2021e). *GreenFILE*. Retrieved August 20, 2021e, from Paywalled URL.

[CR32] EBSCOhost. (2021f). *Medline*. Retrieved August 20, 2021c, from Paywalled URL.

[CR33] EBSCOhost. (2021g). *SocINDEX*. Retrieved August 20, 2021d, from Paywalled URL.

[CR34] EBSCOhost. (2021h). *SPORTDiscus*. Retrieved August 20, 2021e, from Paywalled URL.

[CR35] Education Resources Information Center. (2021). *What is ERIC?* Retrieved August 16, 2021, from https://eric.ed.gov/?faq.

[CR36] Elsevier. (2021). *ScienceDirect*. Retrieved August 20, 2021, from https://www.sciencedirect.com/.

[CR37] Elsevier. (2022a). *Scopus®: Expertly curated abstract & citation database*. Retrieved January 17, 2022, from https://www.elsevier.com/solutions/scopus.

[CR38] Elsevier. (2022b). *What are the most used Subject Area categories and classifications in Scopus?* Retrieved January 18, 2022, from https://service.elsevier.com/app/answers/detail/a_id/14882/supporthub/scopus/~/what-are-the-most-frequent-subject-area-categories-and-classifications-used-in/.

[CR39] Epistemonikos. (2021a). *About Epistemonikos database*. Retrieved August 16, 2021a, from https://www.epistemonikos.org/en/about_us/who_we_are.

[CR40] Epistemonikos. (2021b). *Epistemonikos database methods*. Retrieved August 16, 2021b, from https://www.epistemonikos.org/en/about_us/methods.

[CR41] ERIC. (2022). *What is ERIC?* Retrieved January 17, 2022, from https://eric.ed.gov/?faq.

[CR42] Europe PMC. (2021). *About Europe PMC*. Retrieved August 10, 2021, from https://europepmc.org/About.

[CR43] Flanagan GP (2014). Law librarianship scholarship: A survey of publications using Scopus Data. SSRN Electronic Journal.

[CR44] Franceschini F, Maisano D, Mastrogiacomo L (2016). Empirical analysis and classification of database errors in Scopus and Web of Science. Journal of Informetrics.

[CR45] García-Pérez MA (2010). Accuracy and completeness of publication and citation records in the Web of Science, PsycINFO, and Google Scholar: A case study for the computation of h indices in Psychology. Journal of the American Society for Information Science and Technology.

[CR46] Google Scholar. (2022). *Stand on the shoulders of giants*. Retrieved January 17, 2022, from https://scholar.google.com/intl/en/scholar/about.html.

[CR47] Gusenbauer M (2019). Google Scholar to overshadow them all? Comparing the sizes of 12 academic search engines and bibliographic databases. Scientometrics.

[CR48] Gusenbauer M (2021). The age of abundant scholarly information and its synthesis—A time when ‘just google it’ is no longer enough. Research Synthesis Methods.

[CR49] Gusenbauer M, Haddaway NR (2020). Which academic search systems are suitable for systematic reviews or meta-analyses? evaluating retrieval qualities of Google Scholar, PubMed and 26 other resources. Research Synthesis Methods.

[CR50] Gusenbauer M, Haddaway NR (2021). What every Researcher should know about searching—clarified concepts, search advice, and an agenda to improve finding in academia. Research Synthesis Methods.

[CR51] Haddaway NR, Collins AM, Coughlin D, Kirk S (2015). The role of Google Scholar in evidence reviews and its applicability to grey literature searching. PLoS ONE.

[CR52] Harzing A-W (2019). Two new kids on the block: How do Crossref and Dimensions compare with Google Scholar, Microsoft Academic, Scopus and the Web of Science?. Scientometrics.

[CR53] Harzing A-W, Alakangas S (2016). Google Scholar, Scopus and the Web of Science: A longitudinal and cross-disciplinary comparison. Scientometrics.

[CR54] Herzog C, Lunn BK (2018). Response to the letter 'Field classification of publications in Dimensions: A first case study testing its reliability and validity'. Scientometrics.

[CR55] Higgins, J. P. T., Thomas, J., Chandler, J., Cumpston, M., Li, T., Page, M. J., & Welch, V. A. (Eds.). (2020). Cochrane handbook for systematic reviews of interventions version 6.3 (updated February 2022). Cochrane, 2022. Available from https://www.training.cochrane.org/handbook.

[CR56] Hug SE, Braendle MP (2017). The coverage of Microsoft Academic: Analyzing the publication output of a university. Scientometrics.

[CR57] IEEE. (2021). *About IEEE Xplore*. Retrieve August 16, 2021, from https://ieeexplore.ieee.org/Xplorehelp/overview-of-ieee-xplore/about-ieee-xplore.

[CR58] Jayabalasingham, B., Boverhof, R., Agnew, K., & Klein, S. (2019). *Identifying research supporting the United Nations Sustainable Development Goals*. Mendeley Data, V1.

[CR59] John Wiley & Sons. (2022). *Wiley Online Library: 7.5 million articles from over 1,600 journals, at your fingertips*. Retrieved January 17, 2022, from https://onlinelibrary.wiley.com/researchers.

[CR60] Johnson RA, Bhattacharyya GK (2010). Statistics: Principles and methods.

[CR61] JSTOR. (2022). *ABOUT JSTOR*. Retrieved January 17, 2022, from https://about.jstor.org/.

[CR62] Konno K, Pullin AS (2020). Assessing the risk of bias in choice of search sources for environmental meta-analyses. Research Synthesis Methods.

[CR63] Kousha K, Thelwall M (2008). Sources of Google Scholar citations outside the Science Citation Index: A comparison between four science disciplines. Scientometrics.

[CR64] Kousha K, Thelwall M (2020). COVID-19 publications: Database coverage, citations, readers, tweets, news, Facebook walls, Reddit posts. Quantitative Science Studies.

[CR65] Kugley, S., Wade, A., Thomas, J., Mahood, Q., Jørgensen, A. -M. K., Hammerstrøm, K., Sathe, N. (2016). Searching for studies: A guide to information retrieval for Campbell systematic reviews. *Campbell Methods Guides*. 10.4073/cmg.2016.1

[CR66] Lazarus JV, Palayew A, Rasmussen LN, Andersen TH, Nicholson J, Norgaard O (2020). Searching PubMed to retrieve publications on the COVID-19 pandemic: Comparative analysis of search strings. Journal of Medical Internet Research.

[CR67] Lens. (2022). *Scholarly Search and Analysis*. Retrieved January 17, 2022, from https://www.lens.org/.

[CR68] Martín-Martín A, Orduna-Malea E, Delgado López-Cózar E (2018). Coverage of highly-cited documents in Google Scholar, Web of Science, and Scopus: A multidisciplinary comparison. Scientometrics.

[CR69] Martín-Martín A, Orduna-Malea E, Thelwall M, Delgado López-Cózar E (2018). Google Scholar, Web of Science, and Scopus: A systematic comparison of citations in 252 subject categories. Journal of Informetrics.

[CR70] Martín-Martín A, Thelwall M, Orduna-Malea E, Delgado López-Cózar E (2021). Google Scholar, Microsoft Academic, Scopus, Dimensions, Web of Science, and OpenCitations' COCI: A multidisciplinary comparison of coverage via citations. Scientometrics.

[CR71] Meho LI, Yang K (2007). Impact of data sources on citation counts and rankings of LIS faculty: Web of science versus Scopus and Google Scholar. Journal of the American Society for Information Science and Technology.

[CR72] Mendeley. (2022). *Mendeley*. Retrieved January 17, 2022, from https://www.mendeley.com/.

[CR73] Meta. (2022). *Welcome to Meta*. Retrieved January 17, 2022, from https://www.meta.org/.

[CR74] Microsoft Academic. (2021). *FAQ*. Retrieved August 20, 2021, from https://academic.microsoft.com/faq.

[CR75] Moed HF, Bar-Ilan J, Halevi G (2016). A new methodology for comparing Google Scholar and Scopus. Journal of Informetrics.

[CR76] Mongeon P, Paul-Hus A (2016). The journal coverage of Web of Science and Scopus: A comparative analysis. Scientometrics.

[CR77] Moskaleva, O., & Akoev, M. (2019). *Non-English language publications in Citation Indexes—Quantity and quality*. https://arxiv.org/pdf/1907.06499.

[CR79] National Library of Medicine. (2022). *PubMed Overview*. Retrieved January 17, 2022, from https://pubmed.ncbi.nlm.nih.gov/about/.

[CR80] Nicholas D, Boukacem-Zeghmouri C, Rodríguez-Bravo B, Xu J, Watkinson A, Abrizah A (2017). Where and how early career researchers find scholarly information. Learned Publishing.

[CR81] Nussbaumer-Streit B, Klerings I, Dobrescu AI, Persad E, Stevens A, Garritty C (2020). Excluding non-English publications from evidence-syntheses did not change conclusions: A meta-epidemiological study. Journal of Clinical Epidemiology.

[CR82] OCLC Online Computer Library Center. (2022). *What is WorldCat?* Retrieved January 17, 2022, from https://www.worldcat.org/whatis/default.jsp.

[CR83] OECD. (2007). *Revised field of science and technology (FOS) classification in the Frascati manual*.

[CR84] OECD. (2021). *Inflation (CPI) Indicator*. Retrieved January 16, 2022, from https://www.oecd-ilibrary.org/economics/inflation-cpi/indicator/english_eee82e6e-en.

[CR85] OpenAIRE. (2022). *Link research*. Retrieved January 17, 2022, from https://www.openaire.eu/mission-and-vision.

[CR86] Orduna-Malea E, Martín-Martín A, López-Cózar ED (2017). Google Scholar as a source for scholarly evaluation: A bibliographic review of database errors. Revista Española De Documentación Científica.

[CR87] Orduña-Malea, E., Ayllón, J. M., Martín-Martín, A., & Delgado López-Cózar, E. (2014). About the size of Google Scholar: Playing the numbers. *EC3 Working Papers, 18*(23).

[CR88] Orduña-Malea E, Ayllón JM, Martín-Martín A, Delgado López-Cózar E (2015). Methods for estimating the size of Google Scholar. Scientometrics.

[CR89] Orduña-Malea E, Delgado-López-Cózar E (2018). Dimensions: Re-discovering the ecosystem of scientific information. El Profesional De La Información.

[CR90] Paperity. (2022). *Welcome to Paperity: About*. Retrieved January 17, 2022, from https://paperity.org/about/.

[CR91] Perianes-Rodriguez A, Waltman L, van Eck NJ (2016). Constructing bibliometric networks: A comparison between full and fractional counting. Journal of Informetrics.

[CR92] Pranckutė R (2021). Web of Science (WoS) and Scopus: The Titans of bibliographic information in today’s academic world. Publications.

[CR93] ProQuest. (2021a). *ABI/INFORM Global: About*. Retrieved January 16, 2022, from https://proquest.libguides.com/abiinformglobal.

[CR94] ProQuest. (2021b). *Nursing & Allied Health Database*. Retrieved August 20, 2021, from Paywalled URL.

[CR95] ProQuest. (2022a). *ProQuest dissertations & theses global*. Retrieved January 17, 2022a, from https://about.proquest.com/en/products-services/pqdtglobal/.

[CR96] ProQuest. (2022b). *Public health database*. Retrieved January 17, 2022b, from https://about.proquest.com/en/products-services/publichealth/.

[CR97] SAGE. (2022). *Journals*. Retrieved January 17, 2022, from https://us.sagepub.com/en-us/nam/journals.

[CR98] ScienceOpen. (2022). *ScienceOpen: An interactive discovery environment*. Retrieved January 17, 2022, from https://about.scienceopen.com/.

[CR99] scite. (2022). *Frequently Asked Questions*. Retrieved January 17, 2022, from https://scite.ai/coverage-and-comparison.

[CR100] Sen S, Kumar A (2019). Design and analysis of algorithms: A contemporary perspective.

[CR101] Shen, Z., Ma, H., & Wang, K. (2018). A Web-scale system for scientific knowledge exploration. In F. Liu & T. Solorio (Eds.), *Proceedings of ACL 2018, system demonstrations, Melbourne, Australia* (pp. 87–92). Stroudsburg: Association for Computational Linguistics. 10.18653/V1/P18-4015.

[CR102] Shorten J, Seikel M, Ahrberg JH (2005). Why do you still use Dewey?. Library Resources & Technical Services.

[CR103] Shu F, Julien C-A, Zhang L, Qiu J, Zhang J, Larivière V (2019). Comparing journal and paper level classifications of science. Journal of Informetrics.

[CR104] Singh VK, Singh P, Karmakar M, Leta J, Mayr P (2021). The journal coverage of Web of Science, Scopus and Dimensions: A comparative analysis. Scientometrics.

[CR105] Springer Nature. (2022). *SpringerLink*. Retrieved January 17, 2022, from https://link.springer.com/.

[CR106] Taylor & Francis. (2022). *Journal solutions*. Retrieved January 17, 2022, from https://librarianresources.taylorandfrancis.com/product-info/journals/.

[CR107] Vera-Baceta M-A, Thelwall M, Kousha K (2019). Web of Science and Scopus language coverage. Scientometrics.

[CR108] Vieira ES, Gomes JANF (2009). A comparison of Scopus and Web of Science for a typical university. Scientometrics.

[CR109] Visser M, van Eck NJ, Waltman L (2021). Large-scale comparison of bibliographic data sources: Scopus, Web of Science, Dimensions, Crossref, and Microsoft Academic. Quantitative Science Studies.

[CR110] Walpole SC (2019). Including papers in languages other than English in systematic reviews: Important, feasible, yet often omitted. Journal of Clinical Epidemiology.

[CR111] Walters WH (2007). Google Scholar coverage of a multidisciplinary field. Information Processing & Management.

[CR112] Waltman L, van Eck NJ (2012). A new methodology for constructing a publication-level classification system of science. Journal of the American Society for Information Science and Technology.

[CR113] Wang Q, Waltman L (2016). Large-scale analysis of the accuracy of the journal classification systems of Web of Science and Scopus. Journal of Informetrics.

[CR114] Wilder EI, Walters WH (2021). Using conventional bibliographic databases for social science research: Web of Science and Scopus are not the only options. Scholarly Assessment Reports.

[CR115] Wolters Kluwer Health. (2022a). *APA PsycInfo*. Retrieved January 17, 2022a, from https://ospguides.ovid.com/OSPguides/psycdb.htm.

[CR116] Wolters Kluwer Health. (2022b). *CAB Abstracts Database Guide*. Retrieved January 17, 2022b, from https://ospguides.ovid.com/OSPguides/cabadb.htm.

[CR117] Wolters Kluwer Health. (2022c). *Embase: Excerpta Medica Database Guide*. Retrieved January 17, 2022c, from https://ospguides.ovid.com/OSPguides/embase.htm.

[CR118] Wolters Kluwer Health. (2022d). *MEDLINE® 2021 Database Guide*. Retrieved January 17, 2022d, from https://ospguides.ovid.com/OSPguides/medline.htm.

